# The PhoP-Dependent ncRNA Mcr7 Modulates the TAT Secretion System in *Mycobacterium tuberculosis*

**DOI:** 10.1371/journal.ppat.1004183

**Published:** 2014-05-29

**Authors:** Luis Solans, Jesús Gonzalo-Asensio, Claudia Sala, Andrej Benjak, Swapna Uplekar, Jacques Rougemont, Christophe Guilhot, Wladimir Malaga, Carlos Martín, Stewart T. Cole

**Affiliations:** 1 Grupo de Genética de Micobacterias, Facultad de Medicina, Universidad de Zaragoza, Zaragoza, Spain; 2 CIBER de Enfermedades Respiratorias, Instituto de Salud Carlos III, Madrid, Spain; 3 Servicio de Microbiología, Hospital Universitario Miguel Servet, IIS Aragón, Zaragoza, Spain; 4 Ecole Polytechnique Fédérale de Lausanne, Global Health Institute, Station 19, Lausanne, Switzerland; 5 Ecole Polytechnique Fédérale de Lausanne, Bioinformatics and Biostatistics Core Facility, Station 19, Lausanne, Switzerland; 6 Département Mécanismes Moléculaires des Infections Mycobactériennes, Institut de Pharmacologie et Biologie Structural, CNRS, Toulouse, France; 7 Université Paul Sabatier, Toulouse, France; National Institutes of Health, United States of America

## Abstract

The PhoPR two-component system is essential for virulence in *Mycobacterium tuberculosis* where it controls expression of approximately 2% of the genes, including those for the ESX-1 secretion apparatus, a major virulence determinant. Mutations in *phoP* lead to compromised production of pathogen-specific cell wall components and attenuation both *ex vivo* and *in vivo*. Using antibodies against the native protein in ChIP-seq experiments (chromatin immunoprecipitation followed by high-throughput sequencing) we demonstrated that PhoP binds to at least 35 loci on the *M. tuberculosis* genome. The PhoP regulon comprises several transcriptional regulators as well as genes for polyketide synthases and PE/PPE proteins. Integration of ChIP-seq results with high-resolution transcriptomic analysis (RNA-seq) revealed that PhoP controls 30 genes directly, whilst regulatory cascades are responsible for signal amplification and downstream effects through proteins like EspR, which controls Esx1 function, *via* regulation of the *espACD* operon. The most prominent site of PhoP regulation was located in the intergenic region between *rv2395* and *PE_PGRS41*, where the *mcr7* gene codes for a small non-coding RNA (ncRNA). Northern blot experiments confirmed the absence of Mcr7 in an *M. tuberculosis phoP* mutant as well as low-level expression of the ncRNA in *M. tuberculosis* complex members other than *M. tuberculosis*. By means of genetic and proteomic analyses we demonstrated that Mcr7 modulates translation of the *tatC* mRNA thereby impacting the activity of the Twin Arginine Translocation (Tat) protein secretion apparatus. As a result, secretion of the immunodominant Ag85 complex and the beta-lactamase BlaC is affected, among others. Mcr7, the first ncRNA of *M. tuberculosis* whose function has been established, therefore represents a missing link between the PhoPR two-component system and the downstream functions necessary for successful infection of the host.

## Author Summary

One of the best characterized two-component systems in *Mycobacterium tuberculosis* is represented by the PhoPR pair, with PhoR being the transmembrane sensor kinase and PhoP playing an essential part in controlling expression of virulence-associated genes, such as those encoding the ESX-1 secretion apparatus. Previous studies showed that mutations in *phoP* resulted in attenuation in the mouse model of infection, thus providing the basis for the development of a novel live attenuated *Mycobacterium tuberculosis* vaccine carrying a deletion in *phoP* which is today in clinical trials. To thoroughly investigate the role of PhoP in *M. tuberculosis*, we undertook a systems biology approach comprising ChIP-seq and RNA-seq technologies. We demonstrated binding of PhoP to at least 35 targets on the *M. tuberculosis* chromosome and direct impact on expression of 30 genes, while further amplification of the signal is provided by regulators acting downstream. The strongest binding site was located between *rv2395* and *PE*_*PGRS41*, where transcription of the non-coding RNA (ncRNA) Mcr7 was demonstrated. Expression of Mcr7 was found to be restricted to *M. tuberculosis* species and totally silenced in a *phoP* mutant. Genetics and proteomics approaches proved that Mcr7 controls activity of the Twin Arginine (Tat) secretion system, thus modulating secretion of the immunodominant antigen Ag85 complex and the BlaC beta-lactamase.

## Introduction

*Mycobacterium tuberculosis*, the etiologic agent of tuberculosis in humans, is arguably the world's most important intracellular pathogen. The bacterium disseminates via the aerosol route from open pulmonary lesions of an infectious case and on reaching the alveoli of a susceptible individual is phagocytosed by resident macrophages. After infection, the tubercle bacillus resides asymptomatically for years in 95% of the infected persons. This latent state can be life-long but disease develops when the immune system weakens as a consequence of HIV co-infection, aging or malnutrition [1]. *M. tuberculosis* encounters a variety of environmental conditions and has to adapt to both the extracellular milieu and to the intracellular niche in order to survive [2]. Improved understanding of how this pathogen fine-tunes gene expression to support active growth and non-replicating persistence, and of how it copes with stresses encountered within the host would not only shed light on bacterial pathogenesis and biology but also aid the design of novel intervention strategies.

Well-known adaptation mechanisms in bacteria include two-component signal transduction systems (TCSS). These consist of a sensor protein that, upon reception of specific signal(s), activates its cognate transcription factor resulting in transcriptional regulation of a defined set of genes. The number of TCSS in *M. tuberculosis* is lower than typically found in bacteria of similar genome size, possibly reflecting the evolution of the bacillus as a human pathogen adapted to a predominantly intracellular environment [3]. Of the 11 TCSS present in *M. tuberculosis* H37Rv, the PhoPR TCSS is essential for virulence [4], as demonstrated in *ex vivo* and *in vivo* infection models, where inactivation of *phoP* led to greatly impaired growth [4], [5]. Consistent with these observations, a single nucleotide polymorphism (S219L) in the DNA binding domain of PhoP, which affected the ability of the regulator to control gene expression of the ESX-1 secretion system, was responsible for the reduced virulence of the attenuated strain H37Ra [6], [7]. Biochemical analyses conducted on the *phoP* mutant revealed that the synthesis of the cell wall components diacyltrehaloses, polyacyltrehaloses and sulfolipids, specific to pathogenic mycobacterial species, was also diminished as compared to wild type *M. tuberculosis*, thus providing an additional mechanism for attenuation [8]. Loss of these phenotypes is the basis of candidate vaccine strains carrying deletions in *phoP*, such as the recently developed MTBVAC, which showed great promise upon preclinical evaluation [9].

The PhoP protein is part of the PhoB/OmpR subfamily of transcription factors, characterized by an N-terminal regulatory domain and a DNA binding domain at the C-terminus. The crystal structure revealed the dimeric nature of the regulator and predicted PhoP to bind to direct repeats [10], in agreement with *in vitro* investigations of the PhoP-DNA interactions at a few operator regions [11], [12], [13].

Microarray-based transcriptomic studies of wild type and *phoP* mutant strains have been performed to identify the regulon controlled by PhoP. Genes belonging to lipid and intermediary metabolism, to the PE, PPE and PE_PRGS families and to the transcriptional regulator categories were found to be deregulated in *phoP*-deficient bacteria [5], [14], while a very recent publication [15] identified the genes under direct control of PhoP by means of ChIP-seq experiments on a PhoP-overexpressing strain.

Despite this body of knowledge, several questions remain unanswered. These include defining the external stimulus sensed by the PhoPR TCSS, performing ChIP-seq experiments in physiological conditions, obtaining a single-nucleotide resolution transcriptomic map, and identifying transcriptional regulators acting downstream of PhoP.

In this study, we used a systems biology approach, combining ChIP-seq, RNA-seq and in-depth proteomics, to thoroughly investigate the role of PhoP in the biology of *M. tuberculosis*. We identified a ncRNA, encoded by the *mcr7* gene, as a major target of PhoP and showed its involvement in controlling secretion of Twin Arginine Translocation (Tat) substrates.

## Results

### Genome-wide identification of PhoP binding sites in *M. tuberculosis* by ChIP-seq

The PhoP regulon has been characterized previously by transcription profiling using microarrays in both the H37Rv [5] and MT103 strains [14] of *M. tuberculosis*. Those studies indicated that approximately 2% of genes are regulated by PhoP at the transcriptional level. However, little is known about the biophysical interactions between PhoP and the promoter regions of the genes controlled with the exception of a few well-characterized promoters [13]. Here, we applied ChIP-seq (chromatin immunoprecipitation with anti-PhoP antibodies followed by ultra-high throughput DNA sequencing) to locate PhoP binding sites across the *M. tuberculosis* H37Rv [16] chromosome. To avoid false positive signals we included an isogenic *phoP* mutant [11], which served as a control and reference sample in all experiments.

**Figure 1:**
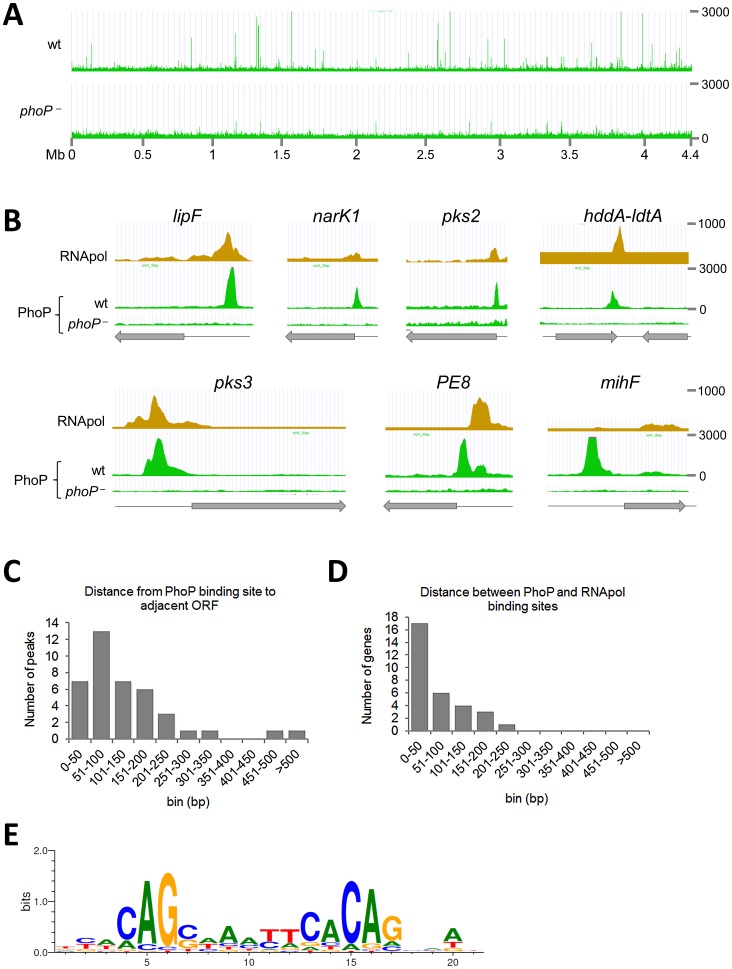
Mapping of PhoP binding sites in the *M. tuberculosis* chromosome by ChIP-seq. **A.** UCSC genome browser view showing PhoP binding regions across the *M. tuberculosis* genome. Peak height correlates with sequence reads and consequently with PhoP binding affinity. The use of a *phoP* mutant in this experiment (bottom panel) allows identification of false positive signals. **B.** PhoP and RNA polymerase (RNApol) ChIP-seq profiles for selected genes. Note that the PhoP binding site lies upstream that of RNApol for *lipF*, *narK1*, *pks2* and *pks3*, known to be transcriptionally activated by PhoP. The position of the PhoP binding region lies downstream that of RNApol for the *PE8* gene. PhoP binding at the 3′-end of *hddA* is shown as an example of non-canonical target. **C.** Bar diagram showing distance (in bp) between the PhoP binding sites and the start codon of adjacent ORFs. **D.** Bar diagram showing distance between the position of the PhoP binding site and the summit of the RNApol peak calculated from ChIP-seq data. **E.** Sequence logo showing the consensus sequence derived from the PhoP binding sites identified by ChIP-seq.

ChIP-seq analysis of cultures grown to exponential phase led to the identification of 35 significantly enriched (p<0.0001, FDR 0.00%) regions in H37Rv compared to the *phoP* mutant (Table 1). Several of these peaks were localized between divergently transcribed open reading frames (ORF) or upstream of validated or predicted operons [17], [18], [19], thus increasing the number of genes potentially affected by PhoP binding directly. These targets were randomly distributed along the *M. tuberculosis* genome (Figure 1A) and predominantly located upstream of ORF (Figure 1B). However, we also observed PhoP binding sites in the 3′-end of ORF, as shown for the *hddA*-*ldtA* genes (Figure 1B). All of the functional categories in which the *M. tuberculosis* ORFs have been grouped were represented in the ChIP-seq results, although clear prevalence of regulatory proteins was observed (12% of the total number of signals as compared to 5% representation in the genome). Remarkably, of the 35 regions detected by ChIP-seq, a number of them had not been described previously [5] as being associated with PhoP-regulated genes (i.e. *mcr7*, *PE27*, *PPE43*, *PE31*, *Rv3778c*, *lpdA*).

**Table 1:** Summary of ChIP-seq results showing localization of PhoP binding sites in the *M. tuberculosis* genome.

Rv number	Gene name	ChIP-seq fold enrichment a ^,^ b	RNA-seq fold change (H37Rv/*ΔphoP*)	Deregulated operons [17], [18], [19] (H37Rv/*ΔphoP*)^c^
**MTB000067**	*mcr7*	162.13	380.48	
**Rv1388**	*mihF*	68.48	1.09	
**Rv1179c** §	*Rv1179c*	45.07	9.81	
**Rv1180** §	*pks3*		68.01	*pks3-pks4-papA3-mmpL10*
**Rv3487c** §	*lipF*	39.05	151.59	
**Rv3488** §	*Rv3488*		1.35	
**Rv3622c** §	*PE32*	34.48	1.08	
**Rv3623** §	*lpqG*		−1.19	
**Rv2329c** §	*narK1*	34.15	83.24	
**Rv2330c** §	*lppP*	34.15	−1.11	
**Rv2331** §	*Rv2331*		55.72	
**Rv3312A**	*Rv3312A*	29.14	165.71	
**Rv2633c**	*Rv2633c*	27.02	16.55	*Rv2633c-Rv2632c*
**Rv1185c**	*fadD21*	26.09	8.16	
**Rv1040c**	*PE8*	24.85	−16.40	*PE8-PPE15*
**Rv2769c**	*PE27*	24.35	1.13	
**Rv0756c** §	*Rv0756c*	21.78	−1.24	
**Rv0757** §	*phoP*		ND	
**Rv3686c**	*Rv3686c*	20.32	24.52	
**Rv0115** £	*hdda*	19.68	2.25	
**Rv2137c** §	*Rv2137*	14.71	2.01	
**Rv2138** §	*lppL*		−1.46	
**Rv3849**	*espR*	14.25	1.43	
**Rv2376c**	*cfp2*	13.79	6.84	
**Rv1535**	*Rv1535*	13.43	1.35	
**Rv2768c**	*PPE43*	12.87	1.28	
**Rv3134c** §	*Rv3134c*	12.19	2.84	
**Rv3135** §	*PPE50*		14.72	
**Rv1184c**	*Rv1184c*	11.33	59.20	
**Rv3825c** §	*pks2*	11.12	72.86	*pks2-papA1-mmpL8*
**Rv3826** §	*fadD23*		1.83	
**Rv3476c** §	*kgtP*	10.89	2.18	
**Rv3477** §	*PE31*		20.01	*PE31-PPE60*
**Rv2588c** §	*yajC*	9.82	1.32	
**Rv2589** §	*gabT*		1.00	
**Rv3778c** §	*Rv3778c*	9.81	2.26	
**Rv3779** §	*Rv3779*		−1.46	
**Rv3501c**	*yrbE4A*	9.70	1.22	
**Rv2920c**	*amt*	9.25	−1.30	
**Rv0251c** §	*hsp*	8.14	−3.16	
**Rv0252** §	*nirB*		17.29	*nirB-nirD*
**Rv3303c** §	*lpdA*	7.98	1.76	
**Rv3304** §	*Rv3304*		1.35	
**Rv3862c** §	*whiB6*	7.76	−4.31	
**Rv3863** §	*Rv3863*		−1.18	
**Rv3767c** §	*Rv3767c*	7.19	10.16	
**Rv3768** §	*Rv3768*		−2.47	
**Rv3415c** §	*Rv3415c*	7.00	−1.00	
**Rv3416** §	*whiB3*		6.05	
**Rv3219**	*whiB1*	6.64	−1.00	
**Rv2904c** §	*rplS*	6.61	−1.10	
**Rv2905** §	*lppW*		2.01	

The table lists regions enriched in immunoprecipitated PhoP-DNA complexes from the H37Rv wild type strain as compared to the *phoP* mutant. Fold change expression values as determined by RNA-seq experiments are also reported for the flanking genes. Predicted or validated operons found to be deregulated in the *phoP* mutant are indicated.

a p value <0.0001.

b FDR 0.00%.

c This column lists the predicted operons (according to [17], [18], [19]), located downstream of PhoP binding sites, that were found to be deregulated in the *phoP* mutant compared to the wild type strain.

§ Region detected is between two genes transcribed in opposite direction.

£ Region located in 3′-end of the gene.

ND: fold change expression levels of *phoP* were not quantified since the gene carries a deletion in the mutant strain.

The distance between the PhoP peak and the ORF start site was calculated for each gene and plotted as reported in Figure 1C. The majority (83%) of the PhoP peaks were between 0 and 200 bp upstream of the ORF start site, with 50% of them within the first 100 bp. Two binding sites were considerably further away from the closer ORF: these were the cases of *lipF* (>500 bp) and *rv1535* (472 bp). Only one case was observed with the PhoP peak lying within the ORF: *rv2137c*, where the summit was located 97 bp downstream of the ATG start codon.

To gain insight into the interplay between PhoP and the transcriptional complex, we compared previous ChIP-seq data of RNA polymerase (RNApol) [17] with the PhoP profile obtained here. We observed that PhoP distribution mirrored that of RNApol at the putative promoter regions (Figure 1B). Closer examination indicated that PhoP binding sites precede those of RNApol. Additional confirmation came from calculation of the distance between the PhoP and RNApol signals, which was between 0 and 100 bp for most of the genes (Figure 1D). This might indicate a role of PhoP in positioning RNApol as a prerequisite for transcriptional control. Exceptionally, the PhoP binding region upstream of PE8 lies downstream of the RNApol binding site (Figure 1B). Curiously, we noticed that some strong PhoP peaks lacked a concomitant RNApol signal as illustrated by *mihF* (Figure 1B).

In order to confirm the ChIP-seq data independently, we quantified a selection of PhoP binding sites from the immunoprecipitated DNA of H37Rv, its *phoP* mutant and a control experiment performed without antibody, obtained from biological replicates. The results validated those obtained in ChIP-seq experiments (Figure S1).

### Identification of the PhoP consensus sequence

We used the MEME suite to identify the PhoP consensus sequence from ChIP-seq signals. Two hundred bp surrounding the summit of the peaks were scanned and a motif was found in 83% of the instances (p-value between 5.88e-09 and 4.40e-05, Figure 1E and Worksheet 1 in Table S1). Additional, though more divergent, copies of the same consensus sequence were found in 11 peaks (31%) (Worksheet 1 in Table S1). Overall, six ChIP-seq signals (*rv1535*, *PPE43*, *lpdA*, *rv3767*, *whiB1* and the region between *yajC* and *gabT*) were not found to be associated with the identified motif, suggesting either higher divergence of the sequences or indirect PhoP binding, i.e. mediated by other proteins. While the 5′ to 3′ orientation of the motif generally corresponded to the orientation of the gene associated with the ChIP-seq signal (22 out of 29 cases), we observed 7 exceptions, the most notable being the well-known PhoP-regulated gene *pks3* [13], [14]. In this case the motif was localized on the opposite strand as compared to the direction of transcription of *pks3*.

### Correlation between PhoP binding and transcriptional regulation

To explore the relationship between binding of PhoP and transcriptional control on the target genes, we performed deep transcriptomic analysis by RNA-seq under the same *in vitro* conditions employed for ChIP-seq. We compared exponentially growing H37Rv wild type to the isogenic *phoP* mutant and quantified gene expression according to the functional categories in the TubercuList database (http://tuberculist.epfl.ch), generating results reported in Worksheet 1 in Table S2. An arbitrary 3-fold threshold was applied to the dataset for further analysis.

Integration of the ChIP-seq and RNA-seq data revealed that 19 PhoP binding sites were associated with altered expression of the flanking gene(s) (Table 1). Since some of these ORFs are part of predicted operons [17], [18], [19], the total number of genes under direct control of PhoP was found to be at least 30 in the experimental conditions tested (Table 1). The region showing the most remarkable affinity for PhoP (162-fold enrichment in ChIP-seq) lay between *rv2395* and *PE_PGRS41*. This signal correlated with the expression of a small transcript (Mcr7) in the intergenic region that was severely affected by deletion of PhoP. This small transcript is further characterized later in this work. Other examples are represented by the operons composed of *pks3-pks4-papA3-mmpL10* and *rv2633c-rv2632c*, which were more expressed in the wild type strain, whereas the *PE8-PPE15* transcriptional unit was induced upon deletion of *phoP*, thus demonstrating the dual role of the regulator. On the contrary, 15 ChIP-seq peaks did not correlate with the presence of deregulated transcripts in their vicinity. The most striking signal in this group was the one upstream of *mihF*.

Deeper inspection of the RNA-seq results uncovered 140 transcripts whose expression underwent changes in the *phoP* mutant (Worksheet 1 in Table S2). Since 30 of these were part of the aforementioned operons, the remaining 110 were likely to be indirectly controlled by PhoP through regulatory cascades. It is worth recalling that PhoP binds upstream of genes encoding several transcriptional regulators (*espR, whiB1, whiB3, whiB6*) that may act downstream. Interestingly, an almost equal proportion of genes was found to be activated (68) or repressed (72) as a consequence of the mutation. Upon clustering these transcripts into functional categories, we observed significant enrichment for the “lipid metabolism” group among the up-regulated genes (p = 0.0016, Fisher's Exact test) and for the “PE/PPE” category among the down-regulated genes (p = 0.0024, Fisher's Exact test). Further discussion of the PhoP regulon will be presented elsewhere.

Independent validation of the RNA-seq data was obtained for a subset of genes by quantitative reverse transcription PCR (qRT-PCR), which confirmed the excellent correlation between high-throughput results and targeted quantification (Figure S1).

### Identification of a small ncRNA as the major PhoP-regulated target

**Figure 2:**
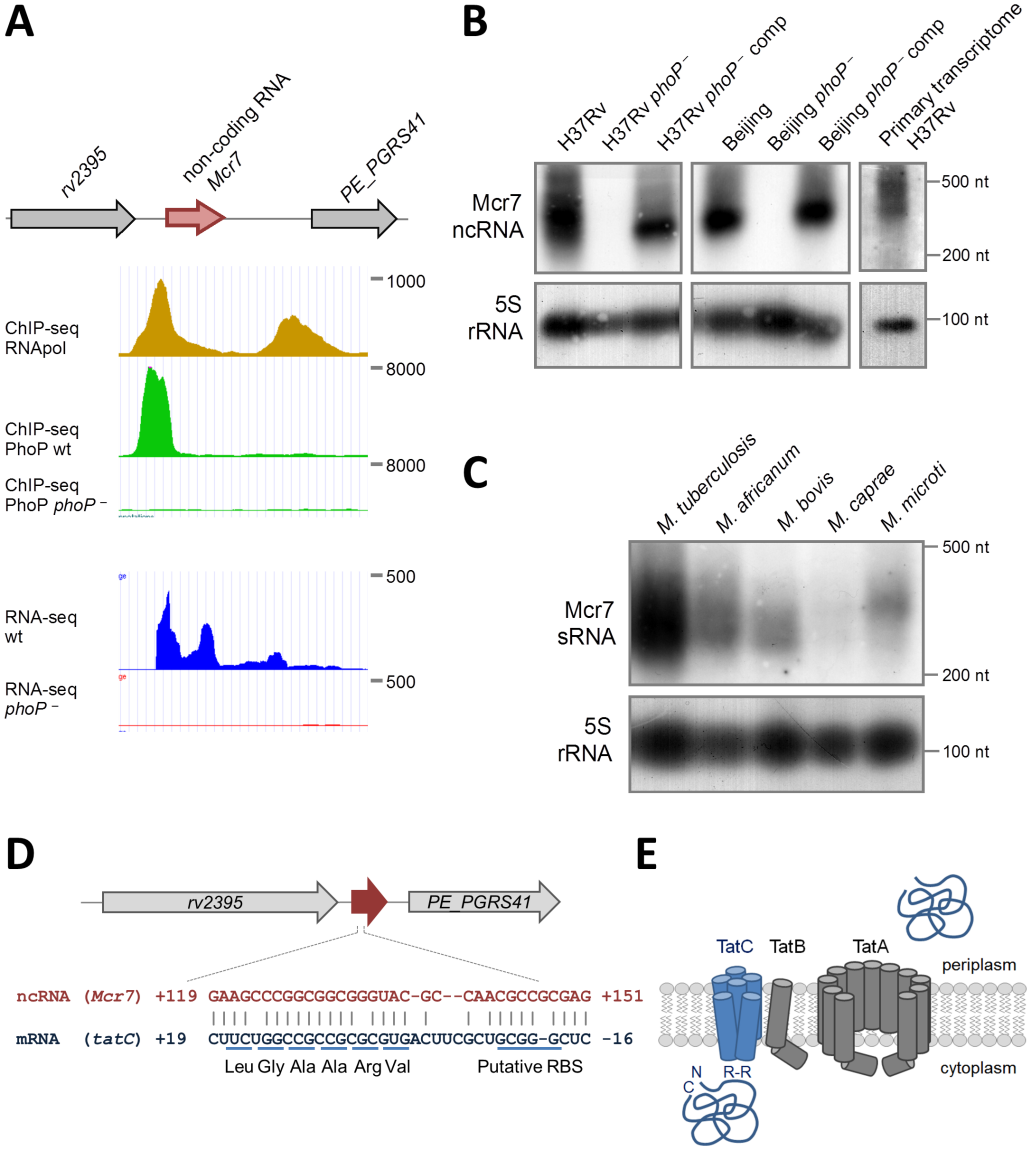
Characterization of *mcr7* as the major PhoP-regulated target. **A.** Diagram showing chromosomal location of *mcr7* in the *rv2395*-*PE*_*PGRS41* intergenic region. ChIP-seq and RNA-seq profiles of the *mcr7* locus showing PhoP binding and transcriptional signals. Note the absence of transcription in the *phoP* mutant that correlates with lack of PhoP-binding in this strain. **B.** Northern blot analysis using a *mcr7* antisense probe in wild type, *phoP* mutant and *phoP*-complemented strains in H37Rv and Beijing GC1237 genetic backgrounds. Detection of the Mcr7 transcript in the primary, unprocessed transcriptome from H37Rv is also shown. Expression of the 5S rRNA is used as a control of RNA loaded in each lane. **C.** Northern blot analysis of Mcr7 in representative species of the *M. tuberculosis* complex. Note the higher expression level of this non-coding RNA in *M. tuberculosis* H37Rv relative to other members of the *M. tuberculosis* complex. Expression of the 5S rRNA is used as a control of RNA loaded in each lane. **D.** Complementarity between Mcr7 and the 5′-end of the *tatC* mRNA. The predicted ribosome binding site (RBS) and positions of the first 6 codons of *tatC* are indicated. **E.** Schematic representation of the Tat system involved in protein translocation from the cytoplasm to the extracellular environment. TatC is involved in recognition of Arg-Arg (RR) motifs within the signal peptide of secreted proteins.

The most prominent PhoP binding site in the genome lay between genes *rv2395* and *PE_PGRS41* (Figure 2A) but, surprisingly, transcription of neither gene differed between strain H37Rv and its *phoP* mutant (Worksheet 1 in Table S2). However, in a previous study of ncRNA in *M. bovis* BCG, the *mcr7* gene encoding a 350 nt transcript, was located within this region [20].

Northern blot analysis was performed on RNA extracted from wild type *M. tuberculosis*, *phoP* mutants and complemented strains in two different genetic backgrounds: the H37Rv laboratory strain and GC1237, a clinical isolate belonging to the Beijing family [21]. We detected an RNA of approximately 350 nt in length in wild type and complemented strains (Figure 2B), whose 5′-end could be mapped from the RNA-seq profile to coordinate 2,692,165 in the H37Rv genome. In contrast, we were unable to identify this RNA in the *phoP* mutants even when 10-times more RNA was used in Northern blot experiments (Figure S2). These results were also confirmed by qRT-PCR showing barely detectable levels of Mcr7 in the *M. tuberculosis phoP* mutant (Figure S2). The complete lack of expression of this ncRNA in the *M. tuberculosis phoP* mutant validates the findings obtained by ChIP-seq and RNA-seq and confirms the *phoP* mutant as an Mcr7-deficient strain as well. Next, we sought to establish whether Mcr7 is a primary transcript or conversely processed from a longer RNA. Detection of an *mcr7* transcript of approximately 350 nt in length in the primary transcriptome of H37Rv (Figure 2B) ruled out the latter possibility and indicated that Mcr7 is a primary, unprocessed RNA.

We then investigated the presence of *mcr7* in the *Mycobacterium* genus by bioinformatic analysis and found that it is predicted to be restricted to the *M. tuberculosis* complex. We therefore obtained expression data for *mcr7* in five representative species. Surprisingly, the Mcr7 ncRNA is only weakly expressed in *M. africanum*, *M. bovis*, *M. caprae* and *M. microti* as compared to the high expression levels in *M. tuberculosis* (Figure 2C).

### Biological function of the Mcr7 ncRNA

After demonstrating the strict PhoP regulation and the predominant expression of *mcr7* in *M. tuberculosis* species, we tried to assign a biological role to this ncRNA. Most *trans*-acting ncRNA act by limited complementarity with their target mRNA, which results in post-transcriptional regulatory mechanisms [22]. A highly structured fold of Mcr7 with a 33-nt free loop (Figure S3) was predicted using the RNAfold server. A bioinformatic search for putative targets of Mcr7 resulted in 18 candidates with complementarity in their 5′-end portion (Figure S3). Given that some ncRNA exert their regulatory function through interaction with their loop structures [23], we focused on mRNAs that annealed with the 33-nt loop. As our previous unpublished results suggested that secretion of Tat-dependent substrates was affected in the *phoP* mutant strain, we focused on the predicted interaction between *tatC* and Mcr7. Interestingly, the 5′-end of the *tatC* mRNA is predicted to base pair with the major loop of Mcr7 (Figure 2D). The interacting region includes the putative ribosome binding site (RBS) and the first 6 codons of the *tatC* mRNA, suggesting that Mcr7 probably prevents ribosome loading and, consequently, translation of *tatC* mRNA. The *tatC* gene is essential for *M. tuberculosis* [24], and encodes a transmembrane protein that is part of the TatABC general secretory apparatus required for export of proteins with a twin arginine motif (RR) in their signal peptide [25] (Figure 2E). TatC recognizes the RR motif prior to protein translocation through the TatA channel (Figure 2E).

### Proteomic analysis shows enrichment of Tat-dependent substrates in the secretome of *M. tuberculosis phoP* mutant lacking *mcr7*

Our prediction suggested that Mcr7 might regulate *tatC* at the post-transcriptional level by occlusion of the RBS and the consequent translational down-regulation (Figure 2D). Consequently, we studied the secretome from exponentially grown cultures of strain H37Rv, its *phoP* mutant and a *phoP* complemented mutant by in-depth proteomics. The enrichment ratio for each protein in the secreted fraction was calculated as the log_2_ of normalized peptide abundance between the desired strains. Results are presented in Worksheet 1 in Table S3. Upon applying a cutoff based on the Significance B value (B<0.05), 37 proteins were found to be more secreted in the *phoP* mutant compared to the wild type strain. Sixteen of these (43.24%) exhibited an RR motif within the first 50 aminoacids. On the contrary, 6 out of 35 proteins, that were more abundant in the wild type displayed the RR motif (17.14%).

**Figure 3:**
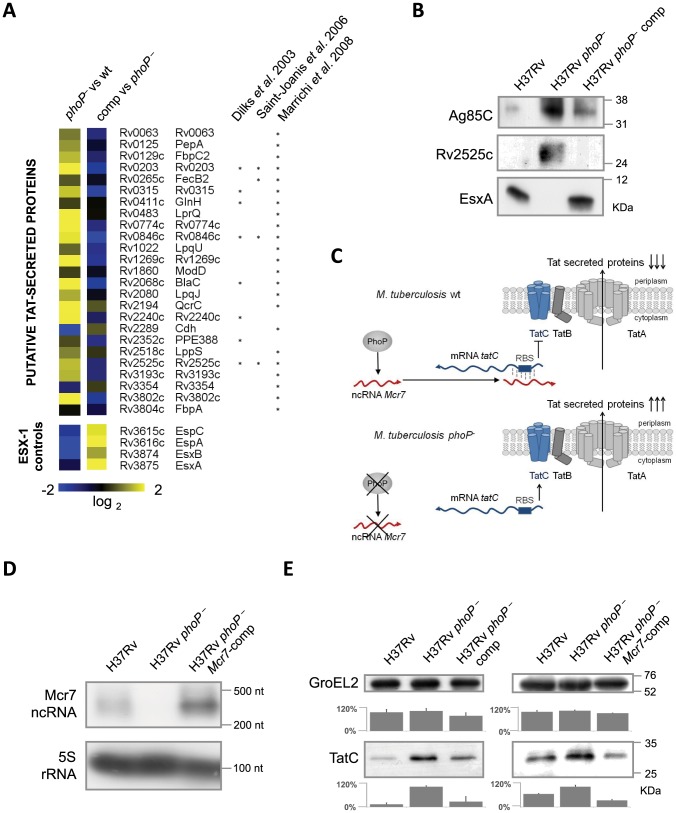
Analysis of the *M. tuberculosis* secretome identifies a high proportion of Tat substrates enriched in the *M. tuberculosis phoP* mutant. **A.** Heat map displaying values from in-depth proteomic quantifications of the secreted protein fraction. Values for EsxA, EsxB, EspA and EspC are displayed as internal controls since these proteins are barely detectable in the *M. tuberculosis phoP* mutant. The other proteins are representative examples of putative Tat-secreted substrates according to [24], [26], [27]. Note the opposite trend between EsxA/EsxB/EspA/EspC and Tat-secreted proteins. **B.** Western blot analysis of Ag85C, Rv2525c and EsxA in *M. tuberculosis* wild type, *phoP* mutant and complemented strains. Results are representative of three independent experiments. **C.** Proposed regulatory network involving PhoP, Mcr7 and *tatC*. PhoP controls transcription of Mcr7 (red transcript), which anneals to the 5′-end of the *tatC* mRNA (blue transcript) occluding the RBS (boxed) and consequently impairing translation of TatC. **D.** Northern blot analysis of Mcr7 in wild type, *phoP* mutant and *mcr7*-complemented strains of *M. tuberculosis*. Introduction of *mcr7* under the 16S rRNA promoter in a *phoP* mutant efficiently restores production of this molecule. Expression of the 5S rRNA is used as a control of RNA loaded in each lane. **E.** Analysis of TatC expression by Western blot in wild type, *phoP* mutant, *phoP*-complemented mutant and *mcr7*-complemented strains of *M. tuberculosis*. Note the higher expression levels of TatC in the *phoP* mutant compared to the wild type. Complementation of a *phoP* mutant with *phoP* or *mcr7* restored wild type levels of TatC. GroEL2 is used as a control of protein loaded in each lane. Signal intensity was quantified from three independent experiments and plotted in the graphs below the Western blot images.

These encouraging findings prompted us to compare the abundance of previously predicted Tat substrates [24], [26], [27] in our secretome experiments. Results indicated that these were significantly more present in the secreted fraction of the *phoP* mutant relative to wild type and complemented strains (Figure 3A). In addition, we compared the relative secretion levels of EsxA (ESAT-6), EsxB (CFP-10), EspA and EspC since these proteins are well-known PhoP-dependent ESX-1 secretion substrates [7] and thus serve as controls. As expected, the secretome of the *phoP* mutant contained very low amounts of EsxA, EsxB, EspA and EspC, thus showing the opposite trend to Tat-dependent substrates (Figure 3A). Next, we validated these results by Western blot analysis of Ag85C [26] and Rv2525c [24] as known Tat-dependent substrates and EsxA as a PhoP-dependent ESX-1 substrate. The results corroborated the proteomic studies: the secreted fraction of the *phoP* mutant showed higher levels of Ag85C and Rv2525c proteins compared to the wild type and complemented mutant strains. On the contrary, EsxA secretion was undetectable in the *phoP* mutant compared to the strains harboring a wild type *phoP* allele (Figure 3B). Taken together, these results are consistent with a regulatory model involving PhoP, Mcr7 and *tatC* mRNA since the absence of Mcr7 in the *phoP* mutant would result in more efficient TatC translation and therefore increased secretion (Figure 3C).

### *Reintroduction* of *mcr7* in an *M. tuberculosis phoP* mutant restores secretion of Tat-dependent proteins to wild type levels

In order to confirm that *mcr7*, but no other PhoP-dependent genes, influenced secretion of Tat substrates *via* post-transcriptional regulation of *tatC* mRNA, we restored Mcr7 production in the *M. tuberculosis phoP* mutant that we have previously demonstrated to be *mcr7* deficient (Figure 2). The *mcr7* gene was cloned downstream of the promoter for the 16S rRNA gene and the resultant construct was integrated into the chromosome of the H37Rv *phoP* mutant, thereby obtaining the *mcr7*-complemented strain. Northern blot experiments confirmed the authenticity and length of the *mcr7* transcript (Figure 3D and Figure S4). Detection of TatC in whole-cell lysates of H37Rv, its *phoP* mutant, the *phoP*-complemented mutant and the *mcr7*-complemented strain demonstrated increased production of this protein in the *phoP* mutant in agreement with our proposed model (Figure 3E). Reintroduction of *phoP* complemented this phenotype as expected. Ectopic expression of *mcr7* in the *phoP* mutant was sufficient to restore TatC levels to the wild type condition (Figure 3E), indicating that *mcr7 per se* was able to modulate expression of TatC, presumably by regulating translation of *tatC* mRNA.

**Figure 4:**
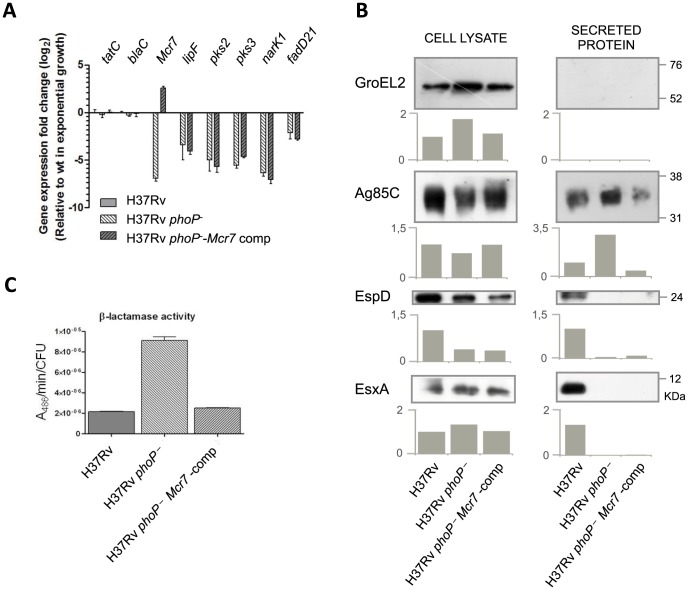
Reintroduction of *mcr7* in an *M. tuberculosis phoP* mutant restores secretion of Tat-dependent substrates to wild type levels. **A.** qRT-PCR analysis of *tatC*, *mcr7* and other genes from the PhoP regulon (*lipF*, *pks2*, *pks3*, *narK1* and *fadD21*). Bars represent fold changes in the expression levels in the *phoP* mutant and *mcr7*-complemented strains relative to *M. tuberculosis* wild type grown exponentially in 7H9 medium. Expression of the *tatC* mRNA is independent of *phoP* mutation and *mcr7* expression. Note that complementation with *mcr7* does not affect expression of the PhoP regulon. Results are representative of three independent experiments and error bars are the standard deviation of the mean. **B.** Western blot experiments of GroEL2, Ag85C, EspD and EsxA in the whole cell and secreted fractions of *M. tuberculosis* wild type, its *phoP* mutant and the *mcr7*-complemented strains. Equal protein amounts were loaded per well. GroEL2 is used as a control of bacterial integrity in each sample. Signal intensity was quantified and plotted in the graphs below the Western blot images. Results are representative of three independent experiments. **C.** Measure of β-lactamase activity of the BlaC protein by nitrocefin chromogenic assay. Activity is calculated relative to the CFU/ml in each strain.

To examine the phenotypic effect caused by reintroducing *mcr7*, we first showed by qRT-PCR that expression of the ncRNA from the surrogate promoter was only about 6-fold higher in the complemented mutant relative to the wild type strain (Figure 4A). Furthermore, we demonstrated that reintroduction of *mcr7* in a *phoP* mutant did not influence the expression of the PhoP regulon that remained at undetectable levels in both the *phoP* mutant and *mcr7*-complemented strains (Figure 4A). Additionally, we proved that transcription of the *tatC* mRNA in the *phoP* mutant and in the *mcr7*-complemented strains showed no significant difference as compared to H37Rv (Figure 4A). Overall, these data ruled out a transcriptional impact of *mcr7* on gene expression and supported the notion of a post-transcriptional effect exerted by the ncRNA on *tatC*.

We then investigated whether reintroduction of *mcr7* in the *phoP* mutant restored secretion of Tat-dependent substrates to wild type levels. Western blot analysis of Ag85C in the whole-cell lysate and secreted fractions showed that while Ag85C is produced at very similar levels in all strains, secretion of this protein was more pronounced in the *phoP* mutant compared to the wild type and *mcr7* complemented strains (Figure 4B). By contrast, inspection of ESX-1 substrates showed no detectable secretion of EsxA and EspD in either the *phoP* mutant or the *mcr7*-complemented mutant strains (Figure 4B). Therefore, the Mcr7 ncRNA did not impact the activity of the ESX-1 secretion apparatus whereas it did affect protein secretion through the Tat system.

Finally, we measured the enzymatic activity of a Tat-dependent substrate, the well-characterized BlaC [28] beta-lactamase using the chromogenic cephalosporin substrate nitrocefin. The results indicated faster reaction kinetics in the *phoP* mutant relative to wild type (Figure 4C), a finding correlated with the protein secretion levels observed in proteomic studies. Again, complementation with *mcr7* was sufficient to successfully restore BlaC activity to wild type levels (Figure 4C). Since no deregulation of *blaC* transcription was observed in the *phoP* mutant (Worksheet 1 in Table S2) and in the *mcr7*-complemented strain (Figure 4A), we attributed this effect to post-transcriptional regulation of TatC by Mcr7.

## Discussion

High-resolution systems biology is helping greatly to unravel the complexities of the *M. tuberculosis* “regulome”. Recent works have uncovered a plethora of ncRNA [29] and reconstructed the hypoxia regulatory network [15] in this pathogen. In this study we integrated data from complementary high-throughput sequencing technologies and obtained extensive knowledge on PhoP-dependent transcriptional regulation in the tubercle bacillus. Specifically, ChIP-seq identified the PhoP binding sites along the *M. tuberculosis* chromosome (Figure 1), whereas strand-specific, single-nucleotide resolution transcriptomic analyses revealed previously unknown features of the PhoP regulatory network *in vitro*. Although good overlap was observed between RNA-seq data and published transcriptomic analyses ([5], see Worksheet 2 in Table S2 for comparison), major progress has been made as compared to traditional microarray-based approaches as indirect regulatory effects present in former studies [5], [14] have been unmasked. Importantly, we found many genes that were deregulated in the *phoP* mutant despite the absence of a PhoP binding signal in the respective promoter regions. In this regard, PhoP was found to control expression of several other regulatory proteins (e.g. EspR, WhiB1, WhiB3, WhiB6), which act in downstream regulatory cascades [30], [31]. Independent confirmation for this conclusion was presented recently in a regulatory model predicting production of acyltrehalose-derived lipids to be coordinated by a PhoP-WhiB3 network *via* regulation of *pks2* and *pks3* [15]. Additional proof was obtained upon comparing the transcriptome of the *phoP* mutant with the predicted binding sites of WhiB1, WhiB3 and WhiB6 (information available at http://www.tbdb.org/). Indeed, the overlap was found to include *rv0996*, *rv1004c*, *rv1040c*, *rv2274c* and *rv3289c* for WhiB1, *rv1040c* for WhiB3, and *rv2396* for WhiB6. Concerning EspR, deregulation of the *espACD* operon was reported in an *espR* knockout strain [32], where a binding site for EspR was demonstrated [30]. Interestingly, PhoP was shown to bind upstream of *lipF* and of *lppL*, where EspR is also present [30], thereby increasing the complexity of the regulatory machinery at these loci. The small ncRNA Mcr7 can also be considered as an intermediate regulator in the PhoP global network, although it likely exerts its function at the post-transcriptional level. We will come back to Mcr7 later in the discussion.

Comparison of ChIP-seq and RNA-seq profiles uncovered several genes associated with a PhoP binding site but whose expression was not altered in a PhoP-deficient strain. We hypothesize that these genes may be subjected to additional layers of regulation or may respond to yet unexplored environmental conditions. This is exemplified by the *mihF* gene, which, despite its upstream PhoP binding site, was not found to be deregulated. Since the signal sensed and the downstream components of the PhoPR two-component system have not yet been completely elucidated, it is conceivable that signal transduction originating from PhoR may involve other factors than PhoP, thereby fine-tuning gene expression in *M. tuberculosis* in different conditions.

Galagan and colleagues recently mapped the binding sites of 50 transcription factors, including PhoP, in *M. tuberculosis* [15] by exploiting a tetracycline-inducible promoter system to overexpress the FLAG-tagged version of the protein of interest and using anti-FLAG antibodies in ChIP-seq experiments. Contrary to their approach, we worked in physiological conditions and performed immunoprecipitation assays using antibodies directed against native PhoP, thus avoiding artifacts due to abnormal expression levels or to biased protein-antibody interaction. In addition, use of the *phoP* mutant allowed false positive signals to be avoided. Interestingly, the number of peaks pinpointed in our work (35) was considerably smaller than that reported in Galagan *et al*. [15], where several signals were detected in intergenic as well as in intragenic regions. This could reflect the different methods employed, since artificial expression of PhoP may have increased binding to low affinity sites. Head-to-head comparison revealed that all but two of the peaks (*yajC-gabT* and *lpdA-rv3304*) identified here were also present in the other study (see Worksheet 2 in Table S1 and Worksheet 3 in Table S1 for detailed comparison).

The position of the PhoP peak with respect to the ORF start site merits discussion. We noticed that in the case of *lipF* and *rv1535*, the binding site was located >400 bp upstream of the translation start codon. This is consistent with previous results of footprinting assays for *lipF* [13] and with the presence of long 5′-UTRs, with presumptive regulatory roles, for *lipF* and *rv1535* in the respective RNA-seq profiles. Interesting observations were made upon alignment of the PhoP and RNApol ChIP-seq profiles. PhoP was located upstream of the enzyme in most cases, suggesting a role as a positive regulator, later confirmed by RNA-seq data. On the contrary, PE8 was the only gene associated with a PhoP signal downstream of the RNApol peak, indicating potential steric hindrance and thus prevention of RNApol progression throughout the coding sequence. ChIP-seq analysis can therefore provide clues as to the role fulfilled by a transcription factor depending on the position of its binding site with respect to RNApol.

Inspection of the PhoP targets uncovered unusual binding sites in the 3′-end of ORF such as the one between *hddA* and *ldtA*. Since this peak is at the end of two convergent genes, it likely corresponds to an unmapped small RNA. Closer inspection of this intergenic region revealed the presence of a novel small transcript downregulated in the *phoP* mutant. Another case is represented by *rv2137c*, where the PhoP interacting region was mapped within the ORF, suggesting an alternative, PhoP-dependent start codon. Indeed, a polypeptide starting at the ATG codon at nucleotide 106, in frame with the currently annotated start site, shows more than 85% identity with the corresponding proteins in all other mycobacteria whose genomes have been sequenced.

The bipartite PhoP consensus sequence derived from ChIP-seq analysis is consistent with the crystal structure of the dimeric PhoP regulator that is predicted to bind to direct repeats [10]. It also agrees with previous footprinting experiments demonstrating binding of PhoP upstream of its own gene [11], [13] and in the promoter regions of *lipF*, *fadD21*, *pks2* [13]. On the other hand, the divergent orientation of the PhoP binding motif relative to the *pks3* gene can be subjected to different interpretations. It could be that the adjacent *rv1179c* is the gene directly controlled by PhoP while *pks3* undergoes indirect regulation. Alternatively, transcriptional regulation in that locus might be independent of directional positioning of the transcription factor.

The last years have witnessed increased attention to ncRNA in prokaryotic organisms, including *Salmonella enterica* [33], *Legionella pneumophila* [34], *Listeria monocytogenes* [35] and *M. tuberculosis* [29]. These molecules have been predicted to exert their function at the post-transcriptional level by modulating translation of RNAs [36]. This process has important implications when bacteria face environmental stresses since it allows faster responses than classical transcriptional regulation. In this study we disclose the Mcr7 ncRNA encoded by the *mcr7* gene, located between *rv2395* and PE_PGRS41 (Figure 2). The latter was described as highly repressed in a *phoP* mutant from microarray experiments by Walters and co-workers [5]. Thanks to the increased resolution provided by RNA-seq, we can now identify the heavily deregulated gene as *mcr7* rather than *PE_PGRS41*. The position of the probes in the microarray assay probably did not allow such precision. A similar observation can be made for a study that characterized the transcriptional differences between the avirulent strain H37Ra and H37Rv [6]. The gene encoding *PE_PGRS41* (and likely the associated intergenic region carrying *mcr7*) was found to be the most highly deregulated. Importantly, H37Ra is a natural mutant in the *phoP* gene since it carries a polymorphism in the DNA binding domain [7], [11], thus indicating that reduced virulence is associated with lack of PhoP activity and impaired expression of the locus encoding *mcr7*. We confirmed this prediction by measuring the expression levels of *mcr7* in H37Ra by qRT-PCR. The ncRNA was found to be poorly detectable as compared to H37Rv (Figure S5).

In the same genomic region of the CDC1551 strain, Abramovitch and colleagues postulated the existence of the *aprABC* locus with *aprC* corresponding to *PE_PGRS41* and *aprA* and *aprB* corresponding to ORFs MT2466 and MT2467 [37]. These ORFs were not predicted in strain H37Rv as neither their codon usage nor positional base composition are typical of true protein coding sequences [16], [38]. The *mcr7* gene completely overlaps the hypothetical *aprA*. A major PhoP binding site precedes the *mcr7* gene but there is none immediately upstream of *PE_PGRS41*. Our results from Northern blot experiments clearly showed one prominent band of approximately 350 nt in length corresponding to Mcr7, that was first described in *M. bovis* BCG [20]. This genomic locus is restricted to species belonging to the *M. tuberculosis* complex, including *M. canettii*, and was not identified in *M. kansasii* and in *M. marinum*, although expression of *mcr7* was found to be particularly prominent in *M. tuberculosis* only. The expression pattern of *mcr7* tallies with the proposed evolutionary pathway of the tubercle bacilli [39]. Indeed, those lineages that evolved from a common *M. tuberculosis*-like ancestor by multiple deletions (*M. africanum*, *M. microti*, *M. caprae* and *M. bovis*) express low-levels of the ncRNA as compared to the *M. tuberculosis* strain. In light of our findings, it is tempting to speculate that modulation of the activity of the Tat secretion system by means of a small RNA has played a role in shaping the adaptation of tubercle bacilli and/or in restricting their host spectrum. We investigated the potential role played by Mcr7 in virulence by performing *ex vivo* and *in vivo* infections. Complementation of the *phoP* mutant with *mcr7* alone did not restore the wild type virulence and the strain was more attenuated than the *phoP* mutant (Figure S6). This phenotype may be related to the ectopic overexpression of Mcr7, which was indeed associated with small colony size (data not shown).

The predicted folding model of Mcr7 revealed the presence of a 33-nt loop with the potential to anneal to three candidate mRNAs: *rv2767c*, *rv2053c* and *tatC* (Figure S3). Since our results provided convincing evidence for increased secretion of the Tat substrates, Ag85A and Ag85C, in *phoP* mutants, we prioritized the study of Tat-dependent secretion. However, we cannot exclude a post-transcriptional impact of Mcr7 on expression of the hypothetical membrane proteins Rv2767c and Rv2053c, although their role in *M. tuberculosis* physiology is questionable since previous proteomic experiments failed to detect them in the total proteome or in cellular subfractions [40], [41], [42], [43]). Proteomic analysis demonstrated that proteins secreted through the Tat system are more abundant in the extracellular fraction of the PhoP-deficient strain (Figure 3). A genetic approach relying on complementation of the *phoP* mutant with *mcr7* proved the involvement of the ncRNA in the regulation of Tat-dependent secretion at the post-transcriptional level while no impact on the amount of mRNA was observed (Figures 3 and 4). This is the first report describing the function of a ncRNA in *M. tuberculosis*. Notably, *M. tuberculosis phoP* mutants display pleiotropic phenotypic effects including impaired secretion of ESX-1 substrates [7], compromised production of sulphatides (SL), diacyltrehaloses (DAT) and polyacyltrehaloses (PAT) [8] and reduced virulence in the macrophage and mouse models of infection [4], [5]. Mcr7 was found to be sufficient to re-establish the wild type phenotype with respect to secretion of Tat substrates whereas ESX-1 substrates were unaffected, thus evoking a specific regulatory cascade where Mcr7 acts downstream of PhoP.

Overall, this work refined the role played by PhoP in control of gene expression in *M. tuberculosis*. A previous study reported that PhoP is involved in the regulation of the ESX-1 secretion system [7] but no direct evidence had been provided so far. Here we uncovered the existence of a novel regulatory cascade composed of at least two regulatory factors, PhoP and EspR, that ultimately controls ESX-1 functions, such as secretion of EsxA, *via* regulation of the *espACD* locus [7], [30]. In addition, we demonstrated a role for the PhoP-dependent ncRNA Mcr7 in Tat-dependent secretion of well-known *M. tuberculosis* antigens, namely the immunodominant Ag85 complex. PhoP could therefore also mediate antigenicity and pathogenesis *via* the Ag85 complex itself and/or through trehalose 6,6-dimycolate, an abundant glycolipid in the mycobacterial cell wall whose biosynthesis is catalyzed by Ag85 proteins. The Ag85 complex is also involved in binding to human fibronectin, important for cell adhesion and invasion [44], [45]. Mcr7 could therefore represent the missing link between PhoP and the downstream processes required for successful infection of the host. Finally, our findings provided a new molecular basis to explain the better protection against tuberculosis conferred by the candidate vaccine strain MTBVAC, which carries a deletion in *phoP* [9]. While the reduced virulence results mainly from abrogation of the ESX-1 secretion system and possibly from lack of complex lipids, its efficacy may be ascribed to improved antigenicity properties following silencing of Mcr7 and the ensuing increase in secretion of Tat substrates such as the Ag85 proteins.

## Materials and Methods

### Ethics statement

All animal work has been conducted according to the national and international guidelines. The protocols for animal handling were previously approved by University of Zaragoza Animal Ethics Committee (protocol number PI43/10).

### Bacterial strains and culture conditions

*Mycobacterium tuberculosis* H37Rv [16], GC1237 [21] wild type strains and their isogenic *phoP* mutants were previously described [11]. The growth rate of the wild type and of the mutant strains were similar (Figure S7). *M. bovis* AF2122/97 [46], *M. caprae* M57 [39], *M. microti* 15496 and *M. africanum* MAF419 [47] were used as representative strains of the *M. tuberculosis* complex. Mycobacterial strains were grown at 37°C in 7H9 medium (Difco) supplemented with 0.05% Tween 80 and 10% albumin-dextrose-catalase (ADC, Middlebrook) or on 7H10 plates supplemented with 10% ADC. For *M. tuberculosis* complex strains different from *M. tuberculosis*, 40 mM sodium pyruvate was added to the medium. *Escherichia coli* DH5α used for cloning procedures was grown at 37°C in LB broth or on LB agar plates. Kanamycin (20 µg/ml) and hygromycin (20 µg/ml) were used as appropriate.

### Chemicals, antibodies and oligonucleotides used in this study

All chemicals were purchased from Sigma-Aldrich, unless otherwise stated. Immunoblotting was performed with mouse monoclonal anti-EsxA antibodies (Hyb 076-08, Abcam), mouse monoclonal anti-Ag85C antibodies (HYT27, Abcam), mouse monoclonal anti-GroEL2 antibodies (BDI578, Abcam), rabbit polyclonal anti-Rv2525c antibodies [24], rat polyclonal anti-EspD antibodies (kindly provided by Jeffrey Chen) and rabbit polyclonal anti-TatC (Eurogentec) antibodies. Polyclonal antibodies to the transcriptional regulator PhoP of *M. tuberculosis* were obtained from rabbits that received five doses of PhoP (0.5 mg), at weeks 0, 4, 8, 12 and 16, respectively. These anti-PhoP antibodies were validated by ELISA (ZEU-Immunotec Zaragoza, Spain). Sequences of the oligonucleotides used in this study will be provided upon request.

### Plasmid construction

The pAZ31 plasmid was kindly provided by Ainhoa Arbues. The pWM222 plasmid was used for *phoPR* complementation in northern blot experiments and was constructed as follows. A 2.7 kbp region spanning the *phoPR* operon was amplified by PCR using primers (5′-ATACTAGTGGCATCACCCAACGCTTGTT-3′) and (5′-ATACTAGTGGTGAGCCAGCTGATCGG-3′). This PCR product was digested with *Spe*I and subsequently transferred into a pMV361 [48] derivative deleted from the phsp60 promoter. In this construct, the *phoPR* operon is expressed from its native promoter. Plasmid pLZ11 used for *mcr7* expression was constructed by inserting a transcriptional fusion of the *rrs* (16S rRNA) promoter with the *mcr7* transcript following a similar strategy to that described in [29]. This transcriptional fusion was accomplished using an overlapping two-step PCR strategy. Briefly, the *rrs* promoter was PCR amplified using primers rrsOV Fw: GACGTCCCGCAGCTGTCGAGCGCT and rrsOV Rv: GGGCCGCCGGCCCTGCCAGTC TAATACAAATCC. The *mcr7* region was amplified using primers mcr7Ov Fw: GACTGGCAGGGCCGGCGGCCC GACACA and mcr7Ov Rv: AAGCTTCCACCTTCTCGTTACCCGCCTCTG. Both PCR products overlap in 21 bp (underlined nucleotides) and were used as self-templates in a PCR reaction. The entire transcriptional fusion was amplified by PCR using the flanking primers rrsOV Fw and mcr7Ov Rv, digested with *Hind*III *and EcoR*I and introduced between the *Hind*III *and EcoR*I sites of pMV361. The resulting construct was introduced in mycobacteria by electroporation and colonies carrying a chromosome-integrated vector were checked by PCR.

### Chromatin immunoprecipitation experiments

Chromatin immunoprecipitation experiments were performed as previously described [49] with the following modifications. We performed two independent ChIP-seq experiments with the wild type strain H37Rv and one experiment with the control *phoP* mutant. Briefly, *M. tuberculosis* cultures were grown to exponential phase (optical density at 600 nm of 0.4) and cross-linked with 1% formaldehyde for ten minutes at 37°C. Cross-linking was quenched by addition of glycine (125 mM). Cells were then washed twice with Tris-buffered saline (TBS, 20 mM Tris-HCl pH 7.5, 150 mM NaCl), resuspended in 4 ml immunoprecipitation (IP) buffer (50 mM Hepes-KOH pH 7.5, 150 mM NaCl, 1 mM EDTA, 1% Triton X-100, 0.1% sodium deoxycholate, 0.1% SDS, protease inhibitor cocktail from Roche) and sonicated to shear DNA using Bioruptor (Diagenode). Cell debris was removed by centrifugation and the supernatant used in IP experiments. Nucleo-protein extracts were incubated with 50 µl of rabbit polyclonal anti-PhoP antibodies at 4°C for 2 days on a rotating wheel. Complexes were subsequently precipitated with Dynabeads (Dynal, anti-rabbit, Invitrogen) for three hours at 4°C. Beads were washed twice with IP buffer, once with IP buffer plus 500 mM NaCl, once with buffer III (10 mM Tris-HCl pH 8, 250 mM LiCl, 1 mM EDTA, 0.5% Nonidet-P40, 0.5% sodium deoxycholate), once with Tris-EDTA buffer pH 7.5. Elution was performed in 50 mM Tris-HCl pH 7.5, 10 mM EDTA, 1% SDS for 40 minutes at 65°C. Samples were finally treated with RNAse A for one hour at 37°C and cross-links were reversed by incubation for two hours at 50°C and for eight hours at 65°C in 0.5× elution buffer with 50 µg Proteinase K (Eurogentec). DNA was purified by phenol-chloroform extraction and quantified by Nanodrop and Qubit fluorometer according to the manufacturer's recommendations (Invitrogen).

### Library preparation for ChIP-seq analysis and Illumina high-throughput sequencing

DNA fragments obtained from the immunoprecipitation procedure were used for library construction and sequencing with the ChIP-Seq Sample Preparation Kit (Illumina), according to the protocol provided by the manufacturer. One lane per library was sequenced on the Illumina Genome Analyzer IIx at the Lausanne Genomics Technologies Facility using the SR Cluster Generation Kit v2 and SBS 36 Cycle Kit v2. Data were processed with the Illumina Pipeline Software v1.40.

### Genome annotation

All analyses in this study were carried out using the *M. tuberculosis* H37Rv annotation from the TubercuList database (http://tuberculist.epfl.ch/), which includes 4019 protein coding sequences (CDS), 73 genes encoding for stable RNAs, small RNAs and tRNAs. In order to quantify protein occupancy and transcription across the entire genome, 3080 intergenic regions (regions flanked by two non-overlapping CDS) were included, resulting in a total of 7172 features.

### ChIP-seq data analysis

ChIP-seq analysis was performed using the HTSstation pipeline at EPFL (http://htsstation.epfl.ch/). Briefly, the single-ended sequence reads generated from ChIP-seq experiments were aligned to the *M. tuberculosis* H37Rv genome (NCBI accession NC_000962.2) using Bowtie [50] with options “-l 28 -best -strata”. Peaks were analysed using MACS v.1.4 [51] with parameters “-bw 200 -m 10100”. Alignment files were converted to bigWig format for visualization in the UCSC genome browser *Mycobacterium tuberculosis* H37Rv 06/20/1998 Assembly [52]. To determine the level of ChIP-seq enrichment for each feature, an enrichment ratio (ER) was calculated by dividing the read count for the ChIP-seq sample in the wild type strain by the read count for the mutant (control) sample.

PhoP binding site motifs were searched using the MEME Suite (http://meme.nbcr.net/meme/) in sequence regions encompassing 100 bp upstream and 100 bp downstream of the predicted peak summit. Motif sequence logo was obtained using WebLogo3 (http://weblogo.threeplusone.com/).

### RNA extraction

Mycobacterial cultures (one for the wild type strain and one for the *phoP* mutant) were grown to exponential phase (OD_600_ = 0.5-0.6) and pelleted by centrifugation. To minimize RNA degradation bacteria were resuspended in 1 ml RNA Protect Bacteria Reagent (Qiagen), incubated for 5 min at room temperature and then centrifuged. Bacterial pellets were resuspended in 0.4 ml lysis buffer (0.5% SDS, 20 mM NaAc, 0.1 mM EDTA) and 1 ml phenol:chloroform (pH = 4.5) 1∶1. Suspensions were transferred to tubes containing glass beads (Qbiogene) and lysed using a ribolyser (Fast-prep instrument) with a three-cycle program (15 sec at speed 6.5 m) including cooling the samples on ice for 5 min between pulses. Samples were then centrifuged and the homogenate was removed from the beads and transferred to a tube containing chloroform:isoamylalcohol 24∶1. Tubes were inverted carefully before centrifugation and the upper (aqueous) phase was then transferred to a fresh tube containing 0.3 M Na-acetate (pH = 5.5) and isopropanol. Precipitated nucleic acids were collected by centrifugation. The pellets were rinsed with 70% ethanol and air dried before being re-dissolved in RNase-free water. DNA was removed from RNA samples using Turbo DNA free (Ambion) by incubation at 37°C for 1 h. RNA integrity was assessed by agarose gel electrophoresis and absence of contaminating DNA was checked by lack of amplification products after 30 PCR cycles.

Primary, unprocessed RNA from H37Rv was prepared as indicated in [53]. Briefly, 10 µg total RNA were treated with 10 U of Terminal 5′-phosphate dependent Exonuclease (Epicentre) for 24 h at 30°C followed by phenol extraction and isopropanol precipitation. Successful preparation of primary transcriptome was confirmed by lack of 23S/16S rRNA bands in agarose gels.

### Library preparation for RNA-seq analysis and Illumina high-throughput sequencing

100 ng of total RNA were mixed with 5× Fragmentation buffer (Applied Biosystems), incubated for 4 minutes at 70°C and then transferred immediately on ice. RNA was purified using RNAClean XP beads (Beckman Coulter), according to the manufacturer's recommendations, and subsequently treated with Antarctic phosphatase (New England Biolabs). RNA was then re-phosphorylated at the 5′-end with polynucleotide kinase (New England Biolabs) and purified with Qiagen RNeasy MinElute columns. In order to ensure strand-specificity, v1.5 sRNA adapters (Illumina) were ligated at the 5′- and 3′-ends using RNA ligase. Reverse transcription was carried out using SuperScript III Reverse Transcriptase (Invitrogen) and SRA RT primer (Illumina). Twelve cycles of PCR amplification using Phusion DNA polymerase were then performed and the library was finally purified with AMPure beads (Beckman Coulter) as per the manufacturer's instructions. A small aliquot (2.5 µl) was analyzed on Invitrogen Qubit and Agilent Bioanalyzer prior to sequencing on Illumina HiSeq 2000 using the TruSeq SR Cluster Generation Kit v3 and TruSeq SBS Kit v3. Data were processed with the Illumina Pipeline Software v1.82.

### RNA-seq data analysis

The single-ended sequence reads generated from RNA-seq experiments were aligned to the *M. tuberculosis* H37Rv genome (NCBI accession NC_000962.2) using Bowtie2 with default parameters [54]. Read counts for all annotated features were obtained with htseq-count program (http://www-huber.embl.de/users/anders/HTSeq/doc/count.html). Regions where genes overlapped were excluded from counting. Reads spanning more than one feature were counted for each feature. Since the RNA library was strand-specific, the orientation of sequence reads had to correspond to the orientation of annotated features to be counted. Analysis of differential gene expression was carried out using the DESeq package [55].

### Quantitative PCR (qRT-PCR) for ChIP-seq and RNA-seq data validation

One microgram of *M. tuberculosis* RNA was converted to cDNA using SuperScript III Reverse Transcriptase (Invitrogen) according to the manufacturer's recommendations. All PCR primers were designed using Primer Express software (Applied Biosystems). The 10 µl PCR reaction consisted of 1× Sybr Green PCR Master Mix (Applied Biosystems), 0.25 µM of each primer and 1 µl of 1∶10 diluted cDNA or IP DNA from immunoprecipitation reactions. Reactions were carried out in triplicate in an Applied Biosystems StepOnePlus Sequence Detection System (Applied Biosystems) according to the manufacturer's instructions. Melting curves were constructed to ensure that only one amplification product was obtained. In the case of qRT-PCR for RNA-seq data confirmation, normalization was obtained to the number of *sigA* molecules in each sample. Regarding the qPCR for ChIP-seq data validation, the number of target molecules was normalized to the mutant (control) sample, after subtraction of the background represented by the mock-IP (no antibody control).

### Northern blot

Northern blot was performed using the DIG Northern starter kit (Roche) following the manufacturer's recommendations. Briefly, total RNA was separated using denaturing 1% agarose gels in 1× MOPS buffer containing 2% formaldehyde. RNA was transferred by capillary blotting to Hybond-N+ nylon membranes (Amersham) and UV-crosslinked prior to incubation with the desired probe. Digoxigenin (DIG)-labelled probes were synthesized to detect *rrf* (5S rRNA) and *mcr7* transcripts using the primer pairs NB-5S-rRNA-fw (ttacggcggccacagcgg)/NB-T7-5S-rRNA-rv (taatacgactcactatagggtgtcctacttttccacccggagggg), NB-mcr7-fw (ccggcggcccgacacatg)/NB-T7-mcr7-rv (taatacgactcactatagggacccgctcaagcaggtcg) respectively. The T7 promoter used for *in vitro* transcription and labeling of RNA is underlined. RNA transcripts complementary to each probe were detected by Western-blot using an anti-DIG antibody conjugated to alkaline phosphatase and the chemiluminescent substrate CDP-*Star*.

### Prediction of Mcr7 interactors

The secondary structure fold of mcr7 was predicted using the RNAfold web server (http://rna.tbi.univie.ac.at/cgi-bin/RNAfold.cgi). Prediction of mcr7 putative targets was performed using TargetRNA (http://cs.wellesley.edu/~btjaden/TargetRNA2/index.html) allowing antisense complementarity from -80 to +20 relative to ORF translation start sites of M. *tuberculosis* H37Rv. A minimum hybridization seed of 7 nt and a p-value threshold of 0.05 were required for target transcripts.

### Preparation of protein samples

In order to avoid albumin contamination in the secreted protein fraction, cultures were grown in 7H9 (Difco) 0.05% Tween 80 supplemented with 0.2% dextrose, 0.085% NaCl. After 2-3 weeks incubation at 37°C, cultures were pelleted by centrifugation. The supernatant containing secreted proteins was incubated with 10% trichloroacetic acid (TCA) for one hour in ice and then centrifuged at 4°C for 30 min. Pelleted proteins were rinsed with cold acetone and then resuspended in 150 mM TrisHCl pH 8. Protein integrity and absence of albumin contamination was checked by SDS-PAGE and Coomassie staining. The pelleted fraction of bacterial cultures was used for extraction of whole-cell proteins. The pellet was resuspended in PBS containing 1% triton ×100 and a cocktail of protease inhibitors (Roche) and sonicated for 30 minutes at 4°C using a Bioruptor (Diagenode). Samples were then centrifuged and the upper phase containing whole-cell lysate was used in downstream experiments. To prepare whole-cell extracts for detection of TatC by Western blot, proteins were further solubilized with 9 M urea, 70 mM DTT and 2% Triton X-100 followed by TCA precipitation and final resuspension in 150 mM TrisHCl pH 8.

### Dimethyl labeling and SAX fractionation

Each sample (8 µg) was reconstituted in 50 µl of 4 M Urea, 10% acetonitrile and buffered with Tris-HCl pH 8.5 to a final concentration of 30 mM. Proteins were reduced using 10 mM dithioerythritol (DTE) at 37°C for 60 min. Cooled samples were subsequently incubated in 40 mM iodoacetamide at 37°C for 45 min in a light-protected environment. Reaction was quenched by addition of DTE to a final concentration of 10 mM. A two-step digestion was performed using Lys-C (1∶50 enzyme: protein) for 2 hours at 37°C. The lysates were first diluted 5-fold and samples were again digested overnight at 37°C using Mass Spectrometry grade trypsin gold (1∶50 enzyme: protein) and 10 mM CaCl_2_. Reaction was stopped by addition of 2 µl of pure formic acid (FA) and peptides were concentrated by vacuum centrifugation to a final volume of 70 µl. Samples were dimethyl-labeled as previously described [56]. The sample H37Rv *phoP^-^* was labeled with light dimethyl reactants (CH_2_O + NaBH_3_CN), the sample H37Rv was labeled with medium reactants (CD_2_O + NaBH_3_CN) and the sample H37Rv *phoP^-^* complemented was labeled with heavy methyl reactants (^13^CD_2_O + NABD_3_CN). As a final step of labeling procedure, samples were mixed in a 1∶1∶1 [(Light: Medium: Heavy) ratio and extensively lyophilized. Technical replicates were obtained. SAX fractionation was performed as previously described [57]. The eluted fractions were dried by vacuum centrifugation and used for LC-MS analysis.

### Mass spectrometry and data analysis

Each SAX fraction was resuspended in 2% acetonitrile, 0.1% FA for LC-MS/MS injections and then loaded on a homemade capillary pre-column (Magic AQ C18; 3 µm by 200 Å; 2 cm×100 µm ID) and separated on a C18 tip-capillary column (Nikkyo Technos Co; Magic AQ C18; 3 µm by 100 Å; 15 cm×75 µm). MS/MS data was acquired in data-dependent mode (over a 4 hr acetonitrile 2–42% gradient) on an Orbitrap Q exactive Mass spectrometer equipped with a Dionex Ultimate 3000 RSLC nano UPLC system and homemade nanoESI source. Acquired RAW files were processed using MaxQuant version 1.3.0.5 [58] and its internal search engine Andromeda [59]. MS/Ms spectra were searched against *M. tuberculosis* strain H37Rv database R23 (http://tuberculist.epfl.ch/) [60]. MaxQuant default identification settings were used in combination with dimethyl-labeling parameters. Search results were filtered with a false-discovery rate of 0.01. Known contaminants and reverse hits were removed before statistical analysis. Relative quantification within different conditions was obtained by calculating the significance B values for each of the identified proteins using Perseus [58].

### Western blotting

Protein samples were quantified using the RC DC protein assay (BioRad) and equal amounts of protein preparations were loaded per well. Proteins were separated on SDS-PAGE 12–15% gels and transferred onto PVDF membranes using a semidry electrophoresis transfer apparatus (Bio-Rad). Membranes were incubated in TBS-T blocking buffer (25 mM Tris pH 7.5, 150 mM NaCl, 0.05% Tween 20) with 5% w/v skimmed milk powder for 30 min prior to overnight incubation with primary antibodies at the dilution indicated below. Membranes were washed in TBS-T three times, and then incubated with secondary antibodies for 1 h before washing. Antibodies were used at the following dilutions: 1∶2,000 for anti-EsxA, 1∶5,000 for anti-Ag85C, 1∶500 for anti-GroEL2, 1∶1,000 for anti-Rv2525, 1∶1,000 for anti-EspD and 1∶1,000 for anti-TatC. Horseradish peroxidase (HRP) conjugated IgG secondary antibodies (Sigma-Aldrich) were used at a 1∶20,000 dilution. Signals were detected using chemiluminescent substrates (GE Healthcare).

### Beta-lactamase assay

Bacterial cultures were grown to OD 600 nm 0.6–0.8 and pelleted. Nitrocefin was added to culture supernatants at 50 mM final concentration and absorbance was measured at 486 nm (Synergy HT BioTEK) every 10 minutes for 3h. Slope of linear range was measured and normalized against total CFUs of the culture.

### J774A.1 infection

Virulence of the different *M. tuberculosis* strains was evaluated in J774A.1 murine macrophages according to a previously published procedure [61], [62]. Briefly, cells were grown in DMEM medium containing 10% fetal bovine serum at 37°C under 5% CO_2_. 10,000 macrophages per well were seeded into a 384-well plate in a total volume of 45 µl and incubated at 37°C for 30 minutes before infection. Cells were infected at an MOI of 10 with titrated stocks of H37Rv, *phoP* mutant, *phoP*-complemented and *mcr7*-complemented strains. On day 3, macrophage survival was measured by exposing the infected cells to PrestoBlue Cell Viability Reagent (Life Technologies) for 1 hour. Fluorescence was read using a TECAN Infinite M200 microplate reader and statistical analysis was performed with the unpaired T-test method.

### Mouse infection

C57BL/6 mice were infected intranasally with an inoculum of 2.5×10^4^ cfu/ml (6 mice per group). Four weeks post-infection mice were euthanized and lungs were plated on 7H11 plates supplemented with 0.5% glycerol, 10% albumin-dextrose-catalase (ADC, Middlebrook), polymixin B 50 U/ml, trimethoprim 0.02 mg/ml and amphotericin B 0.01 mg/ml. Unpaired T-test was used for statistical analysis. The protocols for animal handling were previously approved by University of Zaragoza Animal Ethics Committee (protocol number PI43/10).

### Data access

The ChIP-seq and RNA-seq datasets have been deposited in NCBI's Gene Expression Omnibus [63] under accession number GSE54241.

The mass spectrometry proteomics data have been deposited to the ProteomeXchange Consortium (http://www.proteomexchange.org) via the PRIDE (Proteomics Identification Database) partner repository [64] with the dataset identifier PXD000698.

## Supporting Information

Figure S1 **Validation of ChIP-seq and RNA-seq by qRT-PCR.** **A.** Representative genes from Table 1 showing a significant enrichment (p<0.0001, FDR 0.00%) in H37Rv relative to its *phoP* mutant were independently validated by qRT-PCR. The figure shows absolute quantification using equal amounts of immunoprecipitated material from a control sample without antibody, the H37Rv wild type and its *phoP* mutant. Note the enrichment for the selected regions in the wild type strain compared to the *phoP* mutant and to the sample which was not subjected to incubation with the antibody. **B.** qPCR validation of the peaks detected by ChIP-seq experiments between *hddA* and *ldtA*, upstream of *lipF* and of *rv1535* and within the *rv2137* ORF. Enrichment ratios in the wild type strain as compared to the *phoP* mutant are reported in the graph. **C.** Representative genes showing significant expression differences in RNA-seq (fold change >2 and p<0.05) between H37Rv and the *phoP* mutant were selected for independent validation using qRT-PCR. Figure shows fold change in gene expression in bacteria grown in 7H9 medium. Results are the average of three independent RNA extractions. Error bars indicate the standard deviation of the mean. The *sigA* gene was used as an invariant endogenous control for normalization purposes.
(PDF)

Figure S2 **Detection of the Mcr7 ncRNA by Northern-blot and qRT-PCR.** **A.** Northern blot of the Mcr7 transcript using different RNA amounts of *M. tuberculosis* H37Rv and its *phoP* mutant. Note the absence of transcription of Mcr7 in the mutant even when we used 10 µg RNA/lane. Expression of the 5S rRNA transcript is used as a loading control in each lane. **B.** Fold change in Mcr7 expression in wild type H37Rv relative to the *phoP* mutant calculated by qRT-PCR. Results are the average of three independent RNA extractions. Error bars indicate the standard deviation of the mean. The *sigA* gene was used as an invariant endogenous control for normalization purposes.
(PDF)

Figure S3 **Identification of putative targets of Mcr7.** **A.** Predicted secondary structure of Mcr7. Note the highly structured folding of this non-coding RNA. This secondary structure contains a 33 nt unstructured loop. **B.** Sequence of *mcr7*. The 33 nt loop is labeled in red. **C.** Bioinformatic prediction of putative Mcr7 targets. Annealing position within the *mcr7* sequence is also given. Note that *tatC* and *rv2053c* are the only putative targets that anneal with the unstructured loop of Mcr7.
(PDF)

Figure S4 **Complementation of a** ***M. tuberculosis phoP*** **mutant with** ***mcr7*** **.** **A.** Northern blot analysis using a *mcr7* antisense probe in H37Rv wild type, its *phoP* mutant, the *phoP*-complemented strain and the H37Rv *phoP* mutant complemented with *mcr7*. Expression of the 5S rRNA is used as a control of RNA loaded in each lane. **B.** Quantification of Mcr7 by qRT-PCR. Figure shows fold change in Mcr7 expression relative to the H37Rv *phoP* mutant. The *sigA* gene was used as an invariant endogenous control for normalization purposes. Note that reintroduction of *mcr7* in the H37Rv *phoP* mutant results in transcript length and amount equivalent to those observed in the wild type strain.
(PDF)

Figure S5 **Expression of** ***mcr7*** **in H37Ra.** Relative expression values for *mcr7* in H37Rv, *phoP* mutant and H37Ra were obtained by qRT-PCR. Results are the average of three independent RNA extractions and shown as relative to H37Rv. Error bars indicate the standard deviation of the mean. The *sigA* gene was used as an invariant endogenous control for normalization purposes.
(PDF)

Figure S6 **A. Macrophage infection.** J774A.1 murine macrophages were infected with the strains indicated in the figure at an MOI of 10. Cytotoxicity of the bacterial strains was quantified by measuring fluorescence upon addition of PrestoBlue Cell Viability Reagent (Life Technologies) on day 3 post-infection. (** p<0.0064, *** p<0.0001) **B.** Infection of C57BL/6 mice. Mice were infected via the intranasal route with an inoculum of 2.5×10^4^ cfu/ml (6 mice per group). Four weeks post-infection mice were euthanized and lungs were plated on 7H11 plates supplemented with 0.5% glycerol, 10% albumin-dextrose-catalase (ADC, Middlebrook), polymixin B 50 U/ml, trimetroprim 0.02 mg/ml and amphotericin B 0.01 mg/ml. (** p<0.001, *** p<0.0001).
(PDF)

Figure S7 ***In vitro*** **growth curves of the wild type strain H37Rv and of the** ***phoP*** **mutant.** The growth rates of the wild type strain H37Rv and of the isogenic *phoP* mutant were assessed in 7H9 complete medium at 37°C. Optical density at 600 nm (OD) was recorded and growth curves compiled.
(PDF)

Table S1 **ChIP-seq analysis of the PhoP binding sites in** ***M. tuberculosis*** **H37Rv. Worksheet 1 in Table S1.** List of PhoP binding sites detected by ChIP-seq experiments on the wild type strain H37Rv. The *phoP* mutant strain was used as a negative control to exclude false positive signals. For each peak we provide the following details: peak coordinates (columns B and C), peak length (D), summit coordinate (F), sequence coverage (G), fold enrichment (I), overlap with [15] (L), peak annotation (O). In addition, the summit coordinate for RNApol ChIP-seq [17] is detailed in column M. The motifs identified within each binding site are reported together with the distance from peak summit and the motif orientation. **Worksheet 2 in Table S1 and Worksheet 3 in Table S1.** They include the lists of signals obtained by Galagan and co-workers [15] as they can be downloaded from the TBDB database (http://www.tbdb.org/).
(XLSX)

Table S2 **Differential gene expression analysis between** ***M. tuberculosis*** **H37Rv** ***phoP*** **isogenic mutant and the wild type. Worksheet 1 in Table S2.** This worksheet includes the results obtained upon performing RNA-seq experiments in the wild type strain H37Rv vs. the *phoP* mutant. Sequence coverage is provided in columns B and C for both of the strains for all of the open reading frames (ORF) in the TubercuList database. FoldChange and log2(FoldChange) expression levels are detailed in columns G and H. **Worksheet 2 in Table S2.** This includes the head-to-head comparison between RNA-seq data presented in this manuscript and previous microarray-based transcriptomic analyses by Walters and colleagues [5].
(XLSX)

Table S3 **In-depth proteomic analysis of the secretome of** ***M. tuberculosis*** **H37Rv wild type,** ***phoP*** **mutant and complemented strains. Worksheet 1 in Table S3.** This includes the results obtained upon performing in-depth proteomic analysis of the secreted protein fractions from the wild type strain H37Rv, the *phoP* mutant and the complemented strains. Proteins were identified and labeled according to the annotation in the TubercuList database. Significance B values are detailed in columns I, J, K and L. Details of labeling are provided in the Materials and Methods section and in the Excel workbook.
(XLSX)

## References

[1] Ernst JD (2012) The immunological life cycle of tuberculosis. Nat Rev Immunol 12: 581–591. 22790178 10.1038/nri3259

[2] Russell DG (2011) Mycobacterium tuberculosis and the intimate discourse of a chronic infection. Immunol Rev 240: 252–268. 21349098 10.1111/j.1600-065X.2010.00984.x PMC3174472

[3] Bretl DJ, Demetriadou C, Zahrt TC (2011) Adaptation to environmental stimuli within the host: two-component signal transduction systems of Mycobacterium tuberculosis. Microbiol Mol Biol Rev 75: 566–582. 22126994 10.1128/MMBR.05004-11 PMC3232741

[4] Perez E, Samper S, Bordas Y, Guilhot C, Gicquel B, et al (2001) An essential role for phoP in Mycobacterium tuberculosis virulence. Mol Microbiol 41: 179–187. 11454210 10.1046/j.1365-2958.2001.02500.x

[5] Walters SB, Dubnau E, Kolesnikova I, Laval F, Daffe M, et al (2006) The Mycobacterium tuberculosis PhoPR two-component system regulates genes essential for virulence and complex lipid biosynthesis. Mol Microbiol 60: 312–330. 16573683 10.1111/j.1365-2958.2006.05102.x

[6] Lee JS, Krause R, Schreiber J, Mollenkopf HJ, Kowall J, et al (2008) Mutation in the transcriptional regulator PhoP contributes to avirulence of Mycobacterium tuberculosis H37Ra strain. Cell Host Microbe 3: 97–103. 18312844 10.1016/j.chom.2008.01.002

[7] Frigui W, Bottai D, Majlessi L, Monot M, Josselin E, et al (2008) Control of M. tuberculosis ESAT-6 Secretion and Specific T Cell Recognition by PhoP. PLoS Pathogens 4: e33. 18282096 10.1371/journal.ppat.0040033 PMC2242835

[8] Gonzalo Asensio J, Maia C, Ferrer NL, Barilone N, Laval F, et al (2006) The virulence-associated two-component PhoP-PhoR system controls the biosynthesis of polyketide-derived lipids in Mycobacterium tuberculosis. J Biol Chem 281: 1313–1316. 16326699 10.1074/jbc.C500388200

[9] Arbues A, Aguilo JI, Gonzalo-Asensio J, Marinova D, Uranga S, et al (2013) Construction, characterization and preclinical evaluation of MTBVAC, the first live-attenuated M. tuberculosis-based vaccine to enter clinical trials. Vaccine 31: 4867–4873. 23965219 10.1016/j.vaccine.2013.07.051

[10] Menon S, Wang S (2011) Structure of the response regulator PhoP from Mycobacterium tuberculosis reveals a dimer through the receiver domain. Biochemistry 50: 5948–5957. 21634789 10.1021/bi2005575 PMC3133661

[11] Gonzalo-Asensio J, Soto CY, Arbues A, Sancho J, del Carmen Menendez M, et al (2008) The Mycobacterium tuberculosis phoPR Operon Is Positively Autoregulated in the Virulent Strain H37Rv. Journal of Bacteriology 190: 7068–7078. 18757548 10.1128/JB.00712-08 PMC2580713

[12] Gupta S, Sinha A, Sarkar D (2006) Transcriptional autoregulation by Mycobacterium tuberculosis PhoP involves recognition of novel direct repeat sequences in the regulatory region of the promoter. FEBS Lett 580: 5328–5338. 16979633 10.1016/j.febslet.2006.09.004

[13] Cimino M, Thomas C, Namouchi A, Dubrac S, Gicquel B, et al (2012) Identification of DNA binding motifs of the Mycobacterium tuberculosis PhoP/PhoR two-component signal transduction system. PLoS One 7: e42876. 22880126 10.1371/journal.pone.0042876 PMC3413638

[14] Gonzalo-Asensio J, Mostowy S, Harders-Westerveen J, Huygen K, Hernandez-Pando R, et al (2008) PhoP: a missing piece in the intricate puzzle of Mycobacterium tuberculosis virulence. PLoS One 3: e3496. 18946503 10.1371/journal.pone.0003496 PMC2566814

[15] Galagan JE, Minch K, Peterson M, Lyubetskaya A, Azizi E, et al (2013) The Mycobacterium tuberculosis regulatory network and hypoxia. Nature 499: 178–183. 23823726 10.1038/nature12337 PMC4087036

[16] Cole ST, Brosch R, Parkhill J, Garnier T, Churcher C, et al (1998) Deciphering the biology of Mycobacterium tuberculosis from the complete genome sequence. Nature 393: 537–544. 9634230 10.1038/31159

[17] Uplekar S, Rougemont J, Cole ST, Sala C (2013) High-resolution transcriptome and genome-wide dynamics of RNA polymerase and NusA in Mycobacterium tuberculosis. Nucleic Acids Res 41: 961–977. 23222129 10.1093/nar/gks1260 PMC3553938

[18] Roback P, Beard J, Baumann D, Gille C, Henry K, et al (2007) A predicted operon map for Mycobacterium tuberculosis. Nucleic Acids Res 35: 5085–5095. 17652327 10.1093/nar/gkm518 PMC1976454

[19] Price MN, Huang KH, Alm EJ, Arkin AP (2005) A novel method for accurate operon predictions in all sequenced prokaryotes. Nucleic Acids Res 33: 880–892. 15701760 10.1093/nar/gki232 PMC549399

[20] DiChiara JM, Contreras-Martinez LM, Livny J, Smith D, McDonough KA, et al (2010) Multiple small RNAs identified in Mycobacterium bovis BCG are also expressed in Mycobacterium tuberculosis and Mycobacterium smegmatis. Nucleic Acids Res 38: 4067–4078. 20181675 10.1093/nar/gkq101 PMC2896511

[21] Caminero JA, Pena MJ, Campos-Herrero MI, Rodriguez JC, Garcia I, et al (2001) Epidemiological evidence of the spread of a Mycobacterium tuberculosis strain of the Beijing genotype on Gran Canaria Island. Am J Respir Crit Care Med 164: 1165–1170. 11673204 10.1164/ajrccm.164.7.2101031

[22] Gottesman S, Storz G (2011) Bacterial small RNA regulators: versatile roles and rapidly evolving variations. Cold Spring Harb Perspect Biol 3. 10.1101/cshperspect.a003798 PMC3225950 20980440

[23] Romilly C, Chevalier C, Marzi S, Masquida B, Geissmann T, et al (2012) Loop-loop interactions involved in antisense regulation are processed by the endoribonuclease III in Staphylococcus aureus. RNA Biol 9: 1461–1472. 23134978 10.4161/rna.22710

[24] Saint-Joanis B, Demangel C, Jackson M, Brodin P, Marsollier L, et al (2006) Inactivation of Rv2525c, a substrate of the twin arginine translocation (Tat) system of Mycobacterium tuberculosis, increases beta-lactam susceptibility and virulence. J Bacteriol 188: 6669–6679. 16952959 10.1128/JB.00631-06 PMC1595485

[25] Palmer T, Berks BC (2012) The twin-arginine translocation (Tat) protein export pathway. Nat Rev Microbiol 10: 483–496. 22683878 10.1038/nrmicro2814

[26] Marrichi M, Camacho L, Russell DG, DeLisa MP (2008) Genetic toggling of alkaline phosphatase folding reveals signal peptides for all major modes of transport across the inner membrane of bacteria. J Biol Chem 283: 35223–35235. 18819916 10.1074/jbc.M802660200 PMC3259883

[27] Dilks K, Rose RW, Hartmann E, Pohlschroder M (2003) Prokaryotic utilization of the twin-arginine translocation pathway: a genomic survey. J Bacteriol 185: 1478–1483. 12562823 10.1128/JB.185.4.1478-1483.2003 PMC142874

[28] McDonough JA, Hacker KE, Flores AR, Pavelka MS Jr, Braunstein M (2005) The twin-arginine translocation pathway of Mycobacterium smegmatis is functional and required for the export of mycobacterial beta-lactamases. J Bacteriol 187: 7667–7679. 16267291 10.1128/JB.187.22.7667-7679.2005 PMC1280313

[29] Arnvig KB, Comas I, Thomson NR, Houghton J, Boshoff HI, et al (2011) Sequence-based analysis uncovers an abundance of non-coding RNA in the total transcriptome of Mycobacterium tuberculosis. PLoS Pathog 7: e1002342. 22072964 10.1371/journal.ppat.1002342 PMC3207917

[30] Blasco B, Chen JM, Hartkoorn R, Sala C, Uplekar S, et al (2012) Virulence regulator EspR of Mycobacterium tuberculosis is a nucleoid-associated protein. PLoS Pathog 8: e1002621. 22479184 10.1371/journal.ppat.1002621 PMC3315491

[31] Singh A, Crossman DK, Mai D, Guidry L, Voskuil MI, et al (2009) Mycobacterium tuberculosis WhiB3 maintains redox homeostasis by regulating virulence lipid anabolism to modulate macrophage response. PLoS Pathog 5: e1000545. 19680450 10.1371/journal.ppat.1000545 PMC2718811

[32] Raghavan S, Manzanillo P, Chan K, Dovey C, Cox JS (2008) Secreted transcription factor controls Mycobacterium tuberculosis virulence. Nature 454: 717–721. 18685700 10.1038/nature07219 PMC2862998

[33] Kroger C, Dillon SC, Cameron AD, Papenfort K, Sivasankaran SK, et al (2012) The transcriptional landscape and small RNAs of Salmonella enterica serovar Typhimurium. Proc Natl Acad Sci U S A 109: E1277–1286. 22538806 10.1073/pnas.1201061109 PMC3356629

[34] Sahr T, Bruggemann H, Jules M, Lomma M, Albert-Weissenberger C, et al (2009) Two small ncRNAs jointly govern virulence and transmission in Legionella pneumophila. Mol Microbiol 72: 741–762. 19400772 10.1111/j.1365-2958.2009.06677.x PMC2888818

[35] Toledo-Arana A, Dussurget O, Nikitas G, Sesto N, Guet-Revillet H, et al (2009) The Listeria transcriptional landscape from saprophytism to virulence. Nature 459: 950–956. 19448609 10.1038/nature08080

[36] Gripenland J, Netterling S, Loh E, Tiensuu T, Toledo-Arana A, et al (2010) RNAs: regulators of bacterial virulence. Nat Rev Microbiol 8: 857–866. 21079634 10.1038/nrmicro2457

[37] Abramovitch RB, Rohde KH, Hsu FF, Russell DG (2011) aprABC: a Mycobacterium tuberculosis complex-specific locus that modulates pH-driven adaptation to the macrophage phagosome. Mol Microbiol 80: 678–694. 21401735 10.1111/j.1365-2958.2011.07601.x PMC3138066

[38] Fleischmann RD, Alland D, Eisen JA, Carpenter L, White O, et al (2002) Whole-genome comparison of Mycobacterium tuberculosis clinical and laboratory strains. J Bacteriol 184: 5479–5490. 12218036 10.1128/JB.184.19.5479-5490.2002 PMC135346

[39] Brosch R, Gordon SV, Marmiesse M, Brodin P, Buchrieser C, et al (2002) A new evolutionary scenario for the Mycobacterium tuberculosis complex. Proc Natl Acad Sci U S A 99: 3684–3689. 11891304 10.1073/pnas.052548299 PMC122584

[40] Gu S, Chen J, Dobos KM, Bradbury EM, Belisle JT, et al (2003) Comprehensive proteomic profiling of the membrane constituents of a Mycobacterium tuberculosis strain. Mol Cell Proteomics 2: 1284–1296. 14532352 10.1074/mcp.M300060-MCP200

[41] Mawuenyega KG, Forst CV, Dobos KM, Belisle JT, Chen J, et al (2005) Mycobacterium tuberculosis functional network analysis by global subcellular protein profiling. Mol Biol Cell 16: 396–404. 15525680 10.1091/mbc.E04-04-0329 PMC539182

[42] Malen H, Pathak S, Softeland T, de Souza GA, Wiker HG (2010) Definition of novel cell envelope associated proteins in Triton X-114 extracts of Mycobacterium tuberculosis H37Rv. BMC Microbiol 10: 132. 20429878 10.1186/1471-2180-10-132 PMC2874799

[43] Schubert OT, Mouritsen J, Ludwig C, Rost HL, Rosenberger G, et al (2013) The Mtb proteome library: a resource of assays to quantify the complete proteome of Mycobacterium tuberculosis. Cell Host Microbe 13: 602–612. 23684311 10.1016/j.chom.2013.04.008 PMC3766585

[44] Naito M, Ohara N, Matsumoto S, Yamada T (1998) The novel fibronectin-binding motif and key residues of mycobacteria. J Biol Chem 273: 2905–2909. 9446601 10.1074/jbc.273.5.2905

[45] Kuo CJ, Bell H, Hsieh CL, Ptak CP, Chang YF (2012) Novel mycobacteria antigen 85 complex binding motif on fibronectin. J Biol Chem 287: 1892–1902. 22128161 10.1074/jbc.M111.298687 PMC3265870

[46] Garnier T, Eiglmeier K, Camus JC, Medina N, Mansoor H, et al (2003) The complete genome sequence of Mycobacterium bovis. Proc Natl Acad Sci U S A 100: 7877–7882. 12788972 10.1073/pnas.1130426100 PMC164681

[47] Malaga W, Constant P, Euphrasie D, Cataldi A, Daffe M, et al (2008) Deciphering the genetic bases of the structural diversity of phenolic glycolipids in strains of the Mycobacterium tuberculosis complex. J Biol Chem 283: 15177–15184. 18390543 10.1074/jbc.M710275200 PMC3258887

[48] Stover CK, de la Cruz VF, Fuerst TR, Burlein JE, Benson LA, et al (1991) New use of BCG for recombinant vaccines. Nature 351: 456–460. 1904554 10.1038/351456a0

[49] Sala C, Haouz A, Saul FA, Miras I, Rosenkrands I, et al (2009) Genome-wide regulon and crystal structure of BlaI (Rv1846c) from Mycobacterium tuberculosis. Mol Microbiol 71: 1102–1116. 19154333 10.1111/j.1365-2958.2008.06583.x

[50] Langmead B, Trapnell C, Pop M, Salzberg SL (2009) Ultrafast and memory-efficient alignment of short DNA sequences to the human genome. Genome Biol 10: R25. 19261174 10.1186/gb-2009-10-3-r25 PMC2690996

[51] Zhang Y, Liu T, Meyer CA, Eeckhoute J, Johnson DS, et al (2008) Model-based analysis of ChIP-Seq (MACS). Genome Biol 9: R137. 18798982 10.1186/gb-2008-9-9-r137 PMC2592715

[52] Dreszer TR, Karolchik D, Zweig AS, Hinrichs AS, Raney BJ, et al (2012) The UCSC Genome Browser database: extensions and updates 2011. Nucleic Acids Res 40: D918–923. 22086951 10.1093/nar/gkr1055 PMC3245018

[53] Sharma CM, Hoffmann S, Darfeuille F, Reignier J, Findeiss S, et al (2010) The primary transcriptome of the major human pathogen Helicobacter pylori. Nature 464: 250–255. 20164839 10.1038/nature08756

[54] Langmead B, Salzberg SL (2012) Fast gapped-read alignment with Bowtie 2. Nat Methods 9: 357–359. 22388286 10.1038/nmeth.1923 PMC3322381

[55] Anders S, Huber W (2010) Differential expression analysis for sequence count data. Genome Biol 11: R106. 20979621 10.1186/gb-2010-11-10-r106 PMC3218662

[56] Boersema PJ, Raijmakers R, Lemeer S, Mohammed S, Heck AJ (2009) Multiplex peptide stable isotope dimethyl labeling for quantitative proteomics. Nat Protoc 4: 484–494. 19300442 10.1038/nprot.2009.21

[57] Wisniewski JR, Zougman A, Mann M (2009) Combination of FASP and StageTip-based fractionation allows in-depth analysis of the hippocampal membrane proteome. J Proteome Res 8: 5674–5678. 19848406 10.1021/pr900748n

[58] Cox J, Matic I, Hilger M, Nagaraj N, Selbach M, et al (2009) A practical guide to the MaxQuant computational platform for SILAC-based quantitative proteomics. Nat Protoc 4: 698–705. 19373234 10.1038/nprot.2009.36

[59] Cox J, Neuhauser N, Michalski A, Scheltema RA, Olsen JV, et al (2011) Andromeda: a peptide search engine integrated into the MaxQuant environment. J Proteome Res 10: 1794–1805. 21254760 10.1021/pr101065j

[60] Lew JM, Kapopoulou A, Jones LM, Cole ST (2011) TubercuList—10 years after. Tuberculosis (Edinb) 91: 1–7. 20980199 10.1016/j.tube.2010.09.008

[61] Chen JM, Zhang M, Rybniker J, Basterra L, Dhar N, et al (2013) Phenotypic profiling of Mycobacterium tuberculosis EspA point mutants reveals that blockage of ESAT-6 and CFP-10 secretion in vitro does not always correlate with attenuation of virulence. J Bacteriol 195: 5421–5430. 24078612 10.1128/JB.00967-13 PMC3889621

[62] Kolly GS, Boldrin F, Sala C, Dhar N, Hartkoorn RC, et al (2014) Assessing the essentiality of the decaprenyl-phospho-d-arabinofuranose pathway in Mycobacterium tuberculosis using conditional mutants. Mol Microbiol 92: 194–211. 24517327 10.1111/mmi.12546

[63] Edgar R, Domrachev M, Lash AE (2002) Gene Expression Omnibus: NCBI gene expression and hybridization array data repository. Nucleic Acids Res 30: 207–210. 11752295 10.1093/nar/30.1.207 PMC99122

[64] Vizcaino JA, Cote RG, Csordas A, Dianes JA, Fabregat A, et al (2013) The PRoteomics IDEntifications (PRIDE) database and associated tools: status in 2013. Nucleic Acids Res 41: D1063–1069. 23203882 10.1093/nar/gks1262 PMC3531176

