# Supplementary figures and images for: The PhoP-Dependent ncRNA Mcr7 Modulates the TAT Secretion System in Mycobacterium tuberculosis
Source: PLoS Pathog. 2014 May 29;10(5):e1004183. doi: 10.1371/journal.ppat.1004183 (PMC4038636; doi:10.1371/journal.ppat.1004183)

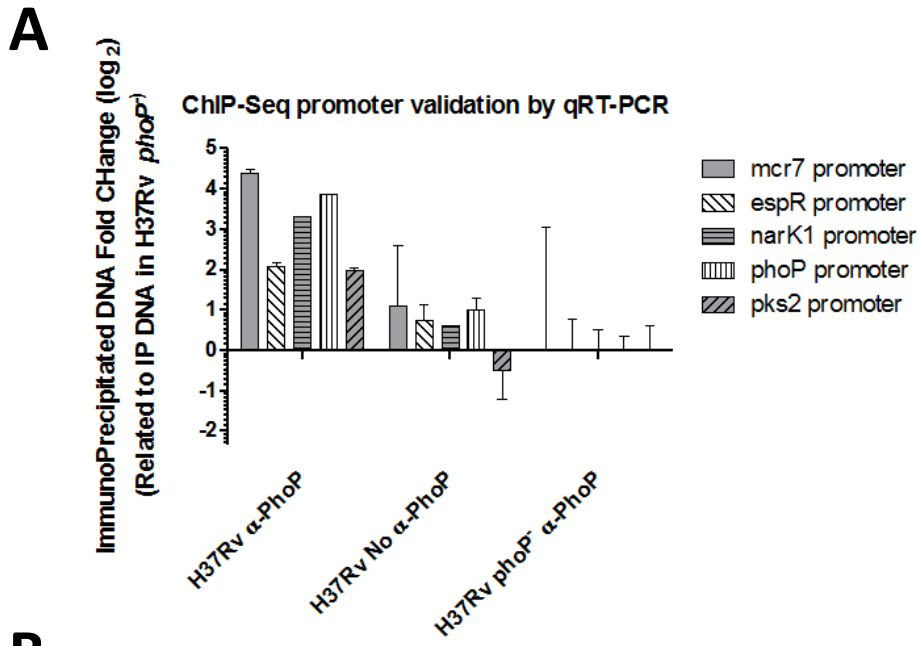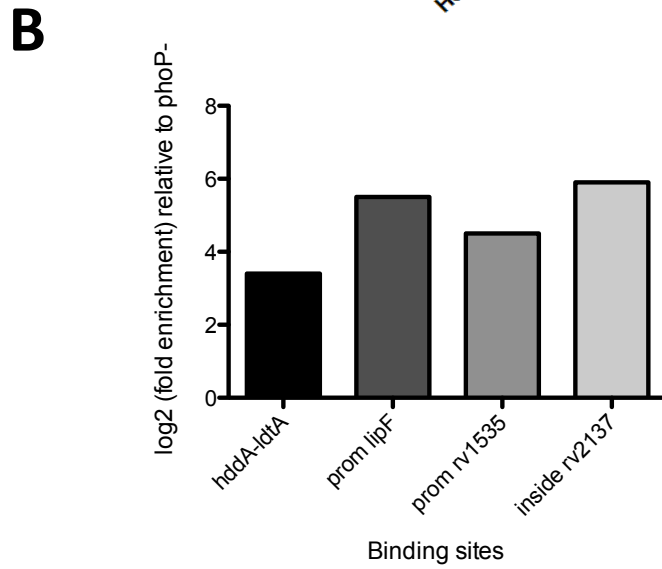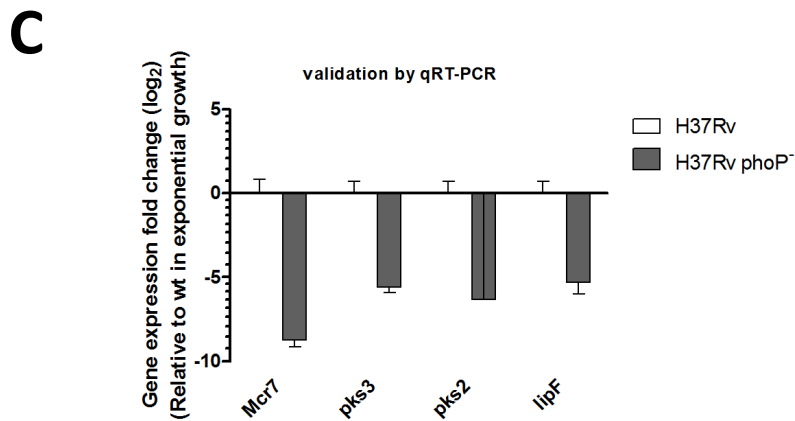

**Figure S1**

Supplement: Figure S1 — Validation of ChIP-seq and RNA-seq by qRT-PCR. A. Representative genes from Table 1 showing a significant enrichment (p<0.0001, FDR 0.00%) in H37Rv relative to its phoP mutant were independently validated by qRT-PCR. The figure shows absolute quantification using equal amounts of immunoprecipitated material from a control sample without antibody, the H37Rv wild type and its phoP mutant. Note the enrichment for the selected regions in the wild type strain compared to the phoP mutant and to the sample which was not subjected to incubation with the antibody. B. qPCR validation of the peaks detected by ChIP-seq experiments between hddA and ldtA, upstream of lipF and of rv1535 and within the rv2137 ORF. Enrichment ratios in the wild type strain as compared to the phoP mutant are reported in the graph. C. Representative genes showing significant expression differences in RNA-seq (fold change >2 and p<0.05) between H37Rv and the phoP mutant were selected for independent validation using qRT-PCR. Figure shows fold change in gene expression in bacteria grown in 7H9 medium. Results are the average of three independent RNA extractions. Error bars indicate the standard deviation of the mean. The sigA gene was used as an invariant endogenous control for normalization purposes. (PDF) [file ppat.1004183.s001.pdf]

**A**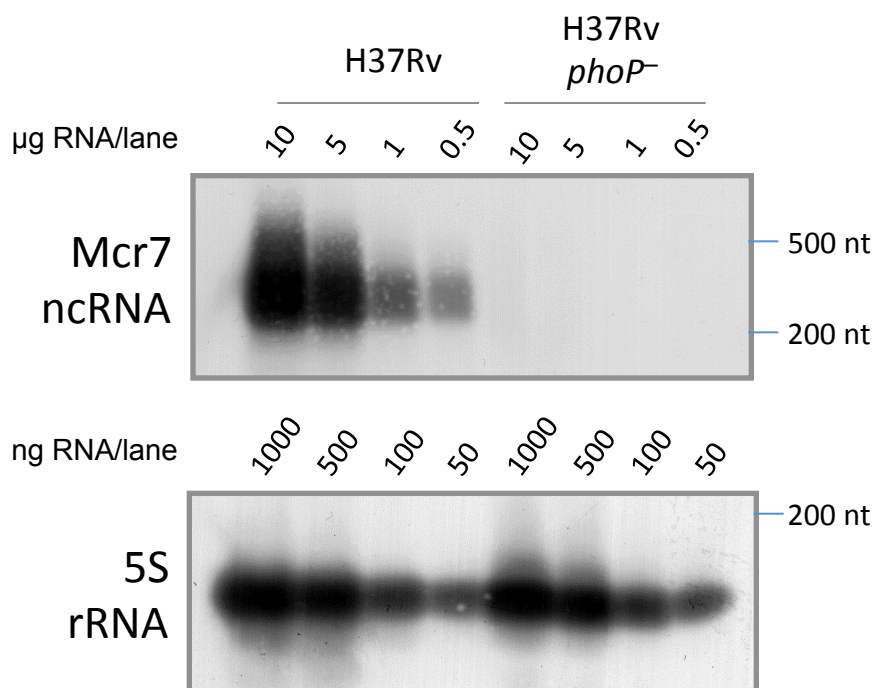**B**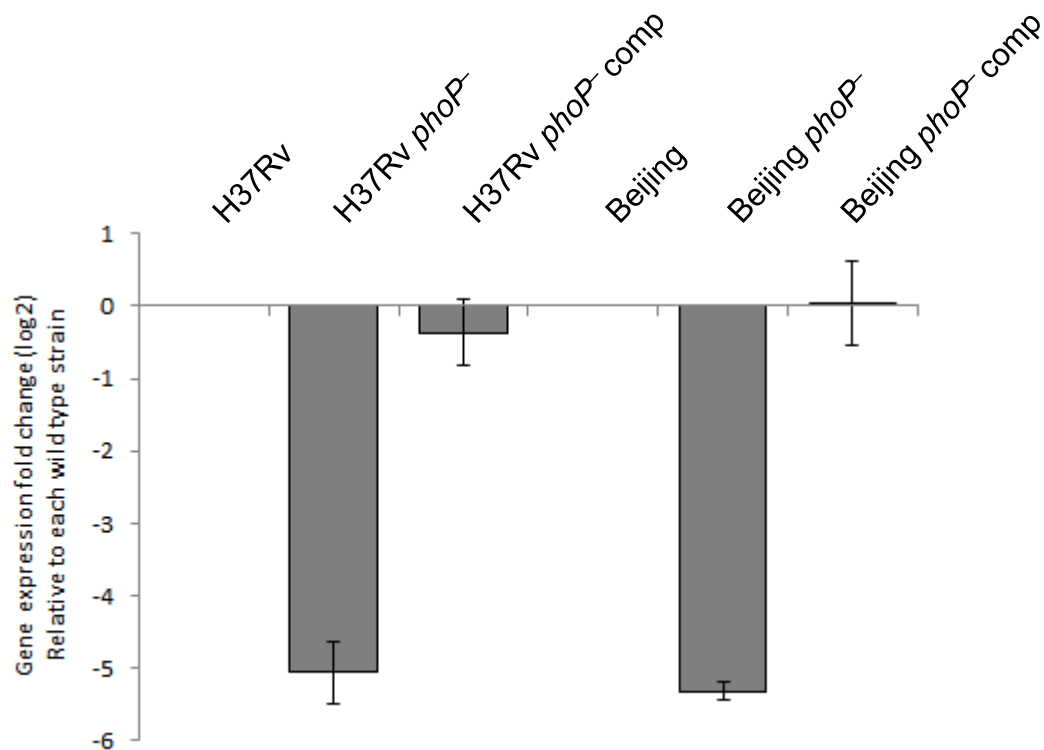**Figure S2**

Supplement: Figure S2 — Detection of the Mcr7 ncRNA by Northern-blot and qRT-PCR. A. Northern blot of the Mcr7 transcript using different RNA amounts of M. tuberculosis H37Rv and its phoP mutant. Note the absence of transcription of Mcr7 in the mutant even when we used 10 µg RNA/lane. Expression of the 5S rRNA transcript is used as a loading control in each lane. B. Fold change in Mcr7 expression in wild type H37Rv relative to the phoP mutant calculated by qRT-PCR. Results are the average of three independent RNA extractions. Error bars indicate the standard deviation of the mean. The sigA gene was used as an invariant endogenous control for normalization purposes. (PDF) [file ppat.1004183.s002.pdf]

**A**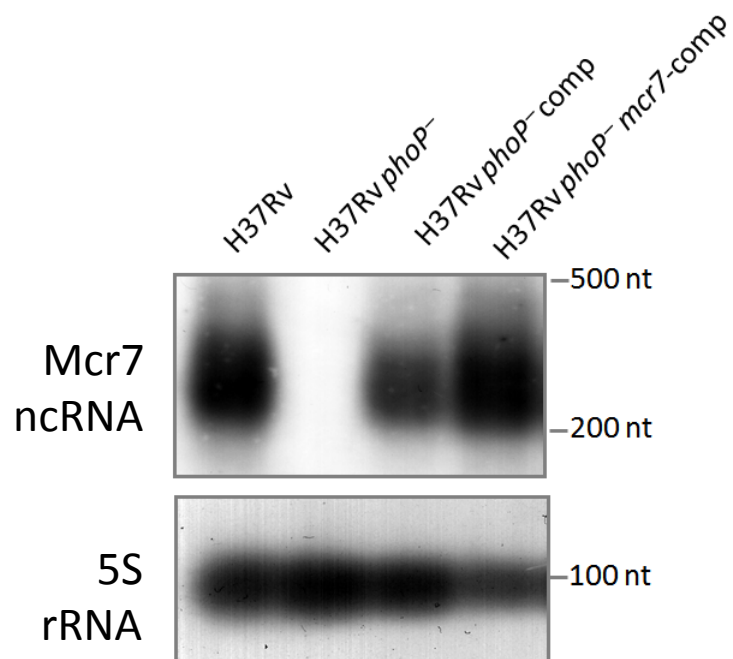**B**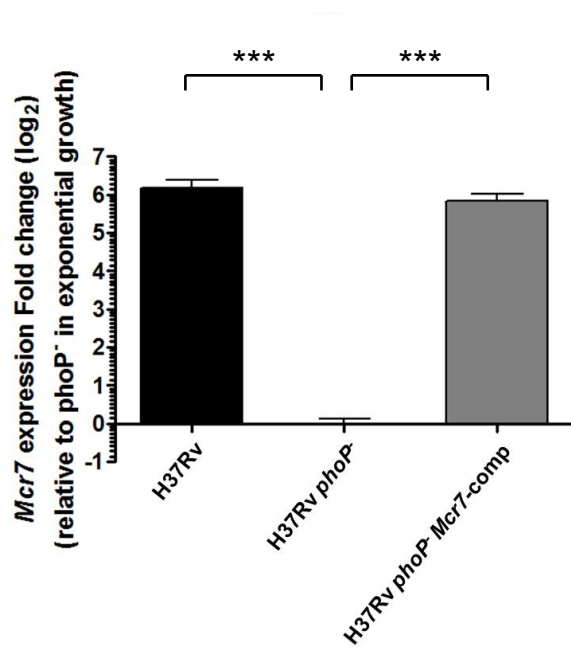**Figure S4**

Supplement: Figure S4 — Complementation of a M. tuberculosis phoP mutant with mcr7 . A. Northern blot analysis using a mcr7 antisense probe in H37Rv wild type, its phoP mutant, the phoP-complemented strain and the H37Rv phoP mutant complemented with mcr7. Expression of the 5S rRNA is used as a control of RNA loaded in each lane. B. Quantification of Mcr7 by qRT-PCR. Figure shows fold change in Mcr7 expression relative to the H37Rv phoP mutant. The sigA gene was used as an invariant endogenous control for normalization purposes. Note that reintroduction of mcr7 in the H37Rv phoP mutant results in transcript length and amount equivalent to those observed in the wild type strain. (PDF) [file ppat.1004183.s004.pdf]

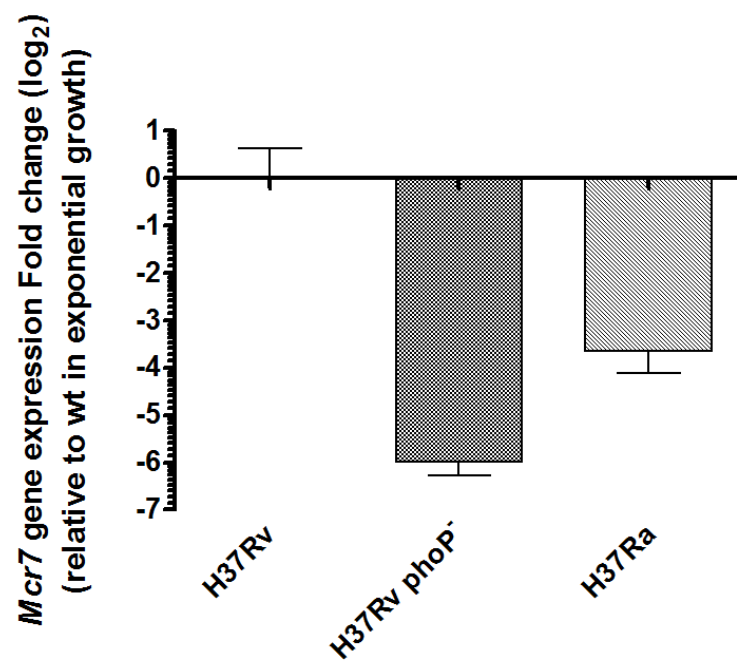

**Figure S5**

Supplement: Figure S5 — Expression of mcr7 in H37Ra. Relative expression values for mcr7 in H37Rv, phoP mutant and H37Ra were obtained by qRT-PCR. Results are the average of three independent RNA extractions and shown as relative to H37Rv. Error bars indicate the standard deviation of the mean. The sigA gene was used as an invariant endogenous control for normalization purposes. (PDF) [file ppat.1004183.s005.pdf]

**A**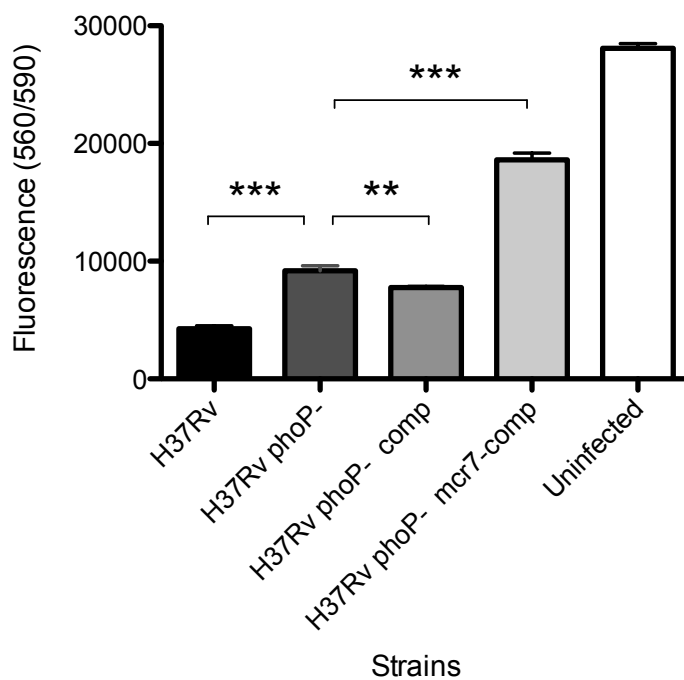**B**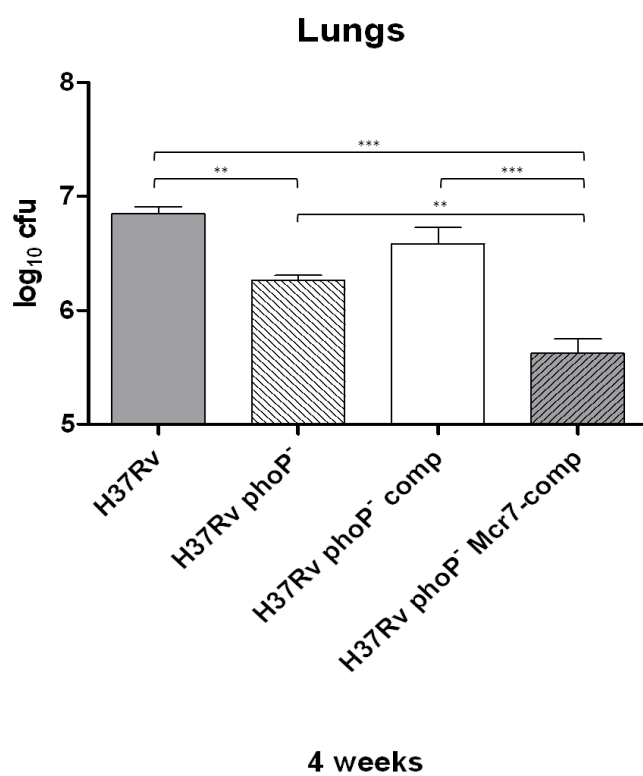**Figure S6**

Supplement: Figure S6 — A. Macrophage infection. J774A.1 murine macrophages were infected with the strains indicated in the figure at an MOI of 10. Cytotoxicity of the bacterial strains was quantified by measuring fluorescence upon addition of PrestoBlue Cell Viability Reagent (Life Technologies) on day 3 post-infection. (** p<0.0064, *** p<0.0001) B. Infection of C57BL/6 mice. Mice were infected via the intranasal route with an inoculum of 2.5×10^4^ cfu/ml (6 mice per group). Four weeks post-infection mice were euthanized and lungs were plated on 7H11 plates supplemented with 0.5% glycerol, 10% albumin-dextrose-catalase (ADC, Middlebrook), polymixin B 50 U/ml, trimetroprim 0.02 mg/ml and amphotericin B 0.01 mg/ml. (** p<0.001, *** p<0.0001). (PDF) [file ppat.1004183.s006.pdf]

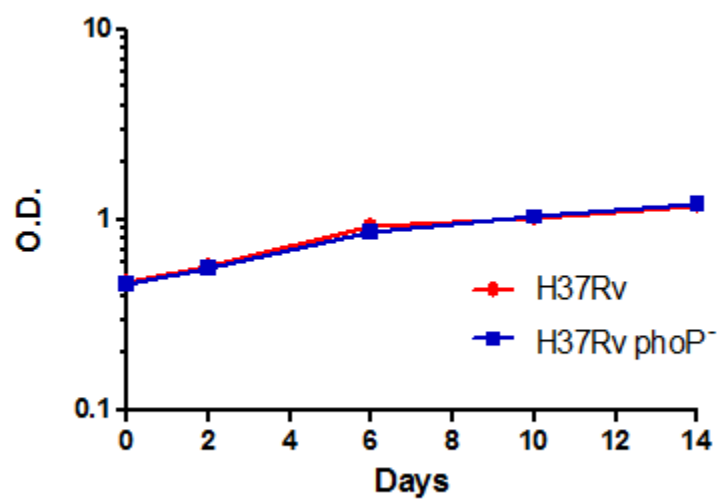

**Figure S7**

Supplement: Figure S7 — In vitro growth curves of the wild type strain H37Rv and of the phoP mutant. The growth rates of the wild type strain H37Rv and of the isogenic phoP mutant were assessed in 7H9 complete medium at 37°C. Optical density at 600 nm (OD) was recorded and growth curves compiled. (PDF) [file ppat.1004183.s007.pdf]
