# Supplementary material for: The PhoP-Dependent ncRNA Mcr7 Modulates the TAT Secretion System in Mycobacterium tuberculosis
Source: PLoS Pathog. 2014 May 29;10(5):e1004183. doi: 10.1371/journal.ppat.1004183 (PMC4038636; doi:10.1371/journal.ppat.1004183)
Supplement: Figure S3 — Identification of putative targets of Mcr7. A. Predicted secondary structure of Mcr7. Note the highly structured folding of this non-coding RNA. This secondary structure contains a 33 nt unstructured loop. B. Sequence of mcr7. The 33 nt loop is labeled in red. C. Bioinformatic prediction of putative Mcr7 targets. Annealing position within the mcr7 sequence is also given. Note that tatC and rv2053c are the only putative targets that anneal with the unstructured loop of Mcr7. (PDF) [file ppat.1004183.s003.pdf]

A

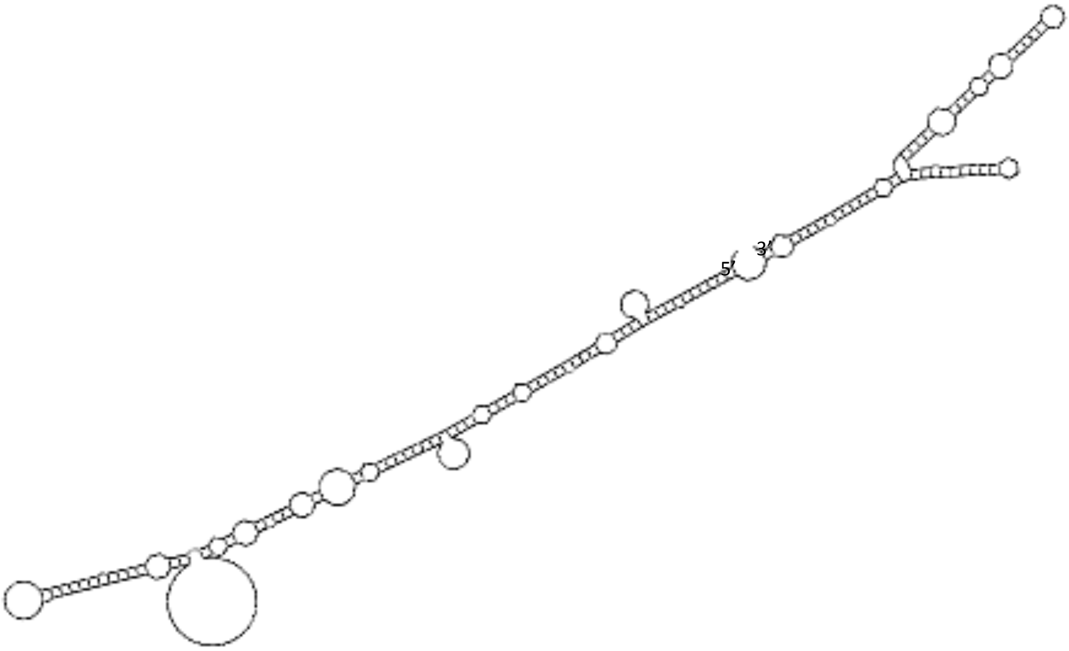

B

5′CCGGCGGCCCGACACAUGCAGCCGAGUUGGCUGGCUCGGAAGGGGACA
GAGUUGACCAUGACAGCGAGUGUGGCCAAGGUGACAGCUGCACGCCCGGAG
CCAAGCGCGGCGUGGGCU<u>GAAGCCCGGCGGCGGGUACGCCAACGCCGCGAG</u>
GACAUGCUGCGCCAUCCUGCAUUUCUGUCCAAGCAGCUCCCUGCCGAACCA
GCAGACGACGACGGCGUCGCGGCCGUCUACGACAUCGCGAUUGCGCGUCGG
CGCCGACCUGCUUGAGCGGGUCCCGGCGGGUCAACGUCGGCGGCUGCCGGG
UAAACCGGCAAUCGACGACCGGGCCUUGGCGGGCGCGUCGCGUUC3′

C

Rank	Gene	Synonym	Score	Pvalue	Annealing position within non-coding RNA
1	Rv2618	Rv2618	-82	0.00157743	
2	ctpA	Rv0092	-81	0.0018378	
3	glnE	Rv2221c	-81	0.0018378	
4	Rv0378	Rv0378	-79	0.0024944	
5	Rv2013	Rv2013	-75	0.00459336	
6	Rv2997	Rv2997	-75	0.00459336	
7	rpsI	Rv3442c	-74	0.00535021	
8	purQ	Rv0788	-73	0.00623137	
9	Rv1751	Rv1751	-73	0.00623137	
10	Rv2733c	Rv2733c	-73	0.00623137	
11	PE_PGRS56	Rv3512	-73	0.00623137	
12	pth	Rv1014c	-72	0.00725713	
13	Rv2767c	Rv2767c	-72	0.00725713	
14	Rv3242c	Rv3242c	-72	0.00725713	
15	tmk	Rv3247c	-72	0.00725713	
16	Rv0698	Rv0698	-71	0.00845103	
17	tatC	Rv2093c	-71	0.00845103	
18	Rv2053c	Rv2053c	-70	0.00984035	

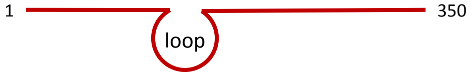

Figure S3
